# Classification of doubly excited molecular electronic states†

**DOI:** 10.1039/d2sc06990c

**Published:** 2023-03-15

**Authors:** Mariana T. do Casal, Josene M. Toldo, Mario Barbatti, Felix Plasser

**Affiliations:** a Aix-Marseille University, CNRS Marseille France; b Institut Universitaire de France 75231 Paris France; c Department of Chemistry, Loughborough University Loughborough LE11 3TU UK f.plasser@lboro.ac.uk

## Abstract

Electronic states with partial or complete doubly excited character play a crucial role in many areas, such as singlet fission and non-linear optical spectroscopy. Although doubly excited states have been studied in polyenes and related systems for many years, the assignment as singly *vs.* doubly excited, even in the simplest case of butadiene, has sparked controversies. So far, no well-defined framework for classifying doubly excited states has been developed, and even more, there is not even a well-accepted definition of doubly excited character as such. Here, we present a solution: a physically motivated definition of doubly excited character based on operator expectation values and density matrices, which works independently of the underlying orbital representation, avoiding ambiguities that have plagued earlier studies. Furthermore, we propose a classification scheme to differentiate three cases: (i) two single excitations occurring within two independent pairs of orbitals leaving four open shells (D_OS_), (ii) the promotion of both electrons to the same orbital, producing a closed-shell determinant (D_CS_), and (iii) a mixture of singly and doubly excited configurations not aligning with either one of the previous cases (D_mix_). We highlight their differences in underlying energy terms and explain their signatures in practical computations. The three cases are illustrated through various high-level computational methods using dimers for D_OS_, polyenes for D_mix_, and cyclobutane and tetrazine for D_CS_. The conversion between D_OS_ and D_CS_ is investigated using a well-known photochemical reaction, the photodimerization of ethylene. This work provides a deeper understanding of doubly excited states and may guide more rigorous discussions toward improving their computational description while also giving insight into their fundamental photophysics.

## Abstract 2

A rigorous classification scheme for doubly excited states is proposed and the different possible types are exemplified in various molecules.

## Graphical abstract

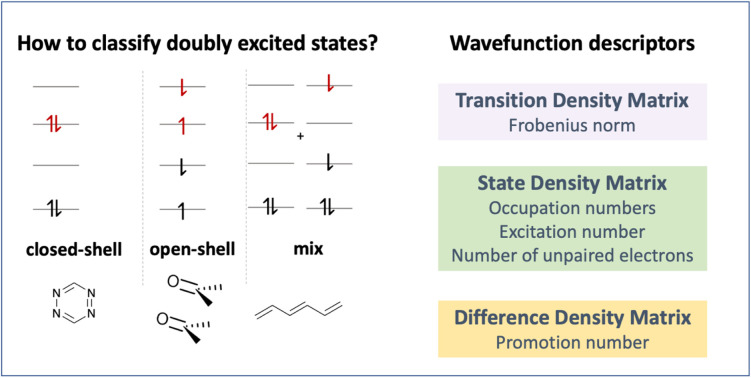

## 1. Introduction

Electronic states with doubly excited characters have aroused interest and generated lively debate in recent years. Their involvement in singlet fission^1–3^ can provide a promising route towards highly efficient photovoltaic devices, but they are also of particular interest in other technological applications, such as non-linear optical spectroscopy,^4–6^ and thermally activated delayed fluorescence.^7^ In the photochemistry of polyenes^8–10^ and derived systems, such as carotenoids,^11,12^ states with partially doubly excited character play a crucial role and have been investigated for over 50 years, yet, still inciting fiery discussions. Despite this substantial interest, there is no well-defined framework for classifying doubly excited states or even a well-accepted definition of doubly excited character. Different authors use different definitions; consequently, even the simple example of the A_g_ state of butadiene, and its assignment as singly or doubly excited, has recently sparked considerable controversy.^13,14^

Indeed, the description of doubly excited states is still a challenge for computational chemistry.^15^ Many commonly used methods, such as linear-response time-dependent density functional theory (TDDFT),^16^ second-order approximate singles and doubles coupled cluster (CC2), or the second-order algebraic diagrammatic construction (ADC(2))^17^ fail in their description. Describing double excitations within a single-reference framework requires going up hierarchies and using more intricate and expensive methods. For example, the CC3 (ref. 18) and ADC(4) methods^19^ are considered appropriate, whereas even ADC(3)^20^ can be problematic.^19,21^ Moreover, in a multireference framework,^22^ one can produce accurate descriptions of doubly excited states. However, these methods are accompanied by the ever-present problems of choosing an appropriate active space and related parameters. Spin-flip methods^23,24^ and state-specific orbital-optimized DFT^25,26^ present themselves as interesting alternatives but require special care in their applications as well. The problem is exacerbated by the fact that there is no clear rule to indicate when such more involved methods are required, and related questions are heavily contested in the literature. A typical example is an ongoing discussion of which methods are suitable to describe the lowest A_g_ state of butadiene.^8,13,14^ Therefore, having a well-defined and method-independent quantifier for double excitation character could greatly help for issues of this type.

Aside from methodological questions, it is also desirable to gain a deeper understanding of the underlying physics of doubly excited states with the eventual goal of designing optimised molecules for specific tasks. At this point, it is particularly interesting to contrast doubly excited states on individual molecules with intermolecular doubly excited states in terms of their energies and wave function properties. However, no rigorous and transferable classification scheme exists that would allow comparing these cases meaningfully. Note that discussions of classification schemes for doubly excited states in the literature are restricted to two-electron atoms where notably different physics is at play.^27,28^

To obtain a basic definition of singly or doubly excited character, one might sum over the weights of all singly excited configurations (denoted %*T*_1_ henceforth). However, such an assignment is only meaningful within a given wave function model and set of reference orbitals. As a consequence, the assignment may vary if a different computational method is chosen, and it is not even immediately clear whether the concept of a doubly excited state possesses intrinsic physical meaning at all (*cf.* ref. 29). More specifically, it is unclear whether any given doubly substituted Slater determinant should be interpreted as contributing to correlation or as an actual double excitation.^13,14^ Furthermore, one should realise from a fundamental physical viewpoint that a double excitation is represented by four correlated particles—two excitation holes and two excited electrons. Formally, such two-body processes should not be described by orbitals but by geminals.^30^ Therefore, significantly enhanced complexity can be expected compared to singly excited states. As a consequence, doubly excited states have remained quite elusive and ambiguous in the discussions so far.

We propose solving the first problem, the assignment of doubly excited states, by using density matrices. They are well-defined independently of the wave function model, thus, allowing us to extract molecular orbital pictures and numerical descriptors from correlated wave functions.^31–33^ More specifically, we base our analysis on transition and difference density matrices, cancelling out correlation contributions also present in the ground state. The use of density matrices provides meaning to these descriptors *via* their connection to physical observables. Specifically, we elaborate on the viewpoint that a doubly (or higher) excited state is a state that cannot be coupled to the ground state with any conceivable one-electron operator (*cf.* ref. 31 and 34). Secondly, to deal with the enhanced complexity of doubly excited states, we combine and contrast several analysis methods to obtain a well-defined and simple yet comprehensive picture. These methods amount to the squared norm of the 1-electron transition density matrix (1TDM),^34,35^ the promotion number^36^ based on the attachment and detachment densities, and the occupation of the natural orbitals (NO), collectively, through the number of unpaired electrons^37^ or, individually, through the occupation of the lowest unoccupied NOs (LUNO and LUNO+1).^38^ In addition, we apply an extension of the excitation number as defined by Barca *et al.*^14^

**Fig. 1:**
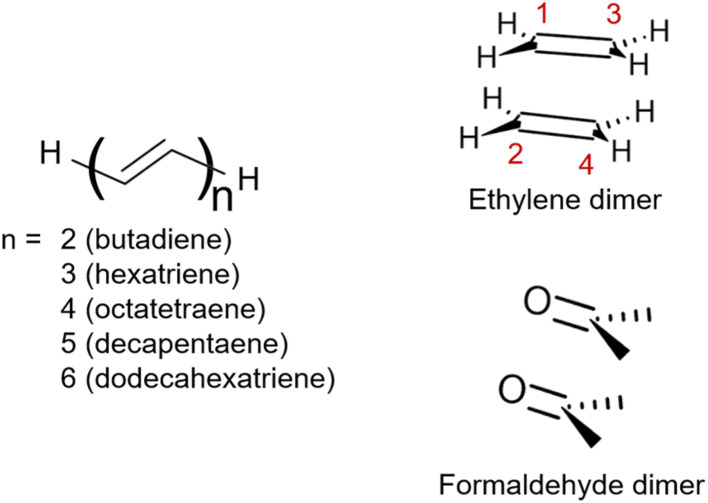
Structures of the molecules studied within this work: the series of all-*trans*-polyenes up to 6 alternating double bounds, ethylene dimer and formaldehyde dimer.

This work aims to comprehensively describe molecular doubly excited states in quantum chemistry computations. We start with a Theory section presenting various definitions of doubly excited character, contrasting different limiting cases for doubly excited states, and discussing the underlying energy contributions. Three illustrative examples follow (Fig. 1). We use the formaldehyde dimer to illustrate the limiting case of a doubly excited state involving two independent pairs of open-shell orbitals (denoted the D_OS_ case). Subsequently, we study polyenes highlighting the complexity of their A_g_ excited states involving a mix of partial doubly and singly excited character (denoted D_mix_). To examine the interconversion between the open- (D_OS_) and closed-shell (D_CS_) limiting cases, we investigate the dimerization of ethylene. Finally, the three different archetypes of doubly excited states are reviewed in a more extended set of molecules.

## 2. Theory

### 2.1 Definition of doubly excited character *via* transition density matrices

Double excitations are traditionally defined *via* the %*T*_1_ values, which reflect the total weight of single excitations. However, the challenge in using %*T*_1_ values is that they are only defined within a given computational method, and it is unclear, for example, how to compare results from single- and multireference computations. Therefore, we choose a different route here. We start with a method-independent definition of doubly excited states based only on physical observables without any explicit reference to orbitals or wave functions. Subsequently, we use this starting point to derive the squared 1TDM norm *Ω* as a rigorous and method-independent substitute for %*T*_1_.

Within the 1TDM picture, we define a state as being doubly (or higher) excited *via* the condition that it is impossible to couple it to the ground state with any conceivable one-electron operator. We can turn this definition into a practical rule if we first realize that an arbitrary transition property of a one-electron operator between wave functions *Ψ*_i_ and *Ψ*_f_ is given as1
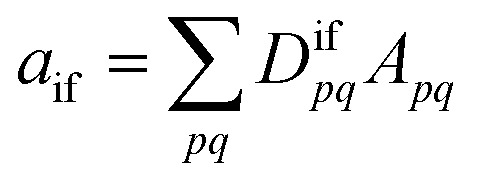
where *D*^if^_*pq*_ is the 1TDM, *A*_*pq*_ is the matrix representation of the operator, and both are given for a molecular orbital (MO) basis {*ϕ*_*p*_}. The 1TDM, in turn, is defined as

2 *D*^if^_*pq*_ = 〈*Ψ*_i_|*p*^†^*q*|*Ψ*_f_〉

where *p*^†^ and *q* are the creation and annihilation operators related to the MOs *ϕ*_*p*_ and *ϕ*_*q*_. Note that *Ψ*_i_ is allowed to be a general, correlated wave function here, meaning that separating these MOs into occupied and virtual orbitals is impossible. Applying the Cauchy–Schwarz inequality to eqn (1),^34^ we obtain3
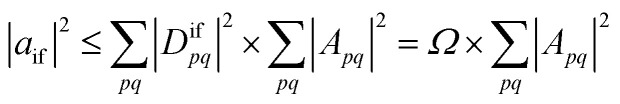
where the symbol *Ω* denotes the squared Frobenius norm of the 1TDM, that is,4
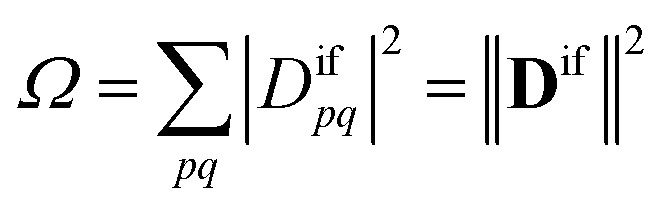


Importantly, we find that the transition property *a*_if_ necessarily vanishes if *Ω* vanishes, that is, if all elements of the 1TDM are zero. Conversely, if any 1TDM element is non-zero, there is at least a conceivable one-electron operator with a non-vanishing transition property. An *Ω* value of zero is equivalent to the statement that the state cannot be coupled *via* a one-electron operator. Thus, a doubly (or higher) excited state exhibits *Ω* = 0, whereas a purely singly excited state exhibits *Ω* = 1.

More generally, *Ω* can be seen as an effective proportionality factor stating how strongly the transition interacts with one-electron operators. Therefore, a value of *Ω* between 0 and 1 can be used to represent a partial doubly excited character.^31^ In practice, the *Ω* value is consistent with the fraction of singly excited amplitudes (%*T*_1_) alluded to above^35^ and presents a natural generalization of this concept. An alternative viewpoint, based on ref. 85, is presented in Section S1.† In Section S2 we discuss the possibility of *Ω* values larger than 1.†

### 2.2 Definition of doubly excited character *via* (difference) density matrices

As an alternative to the 1TDM, it is possible to view doubly excited character *via* the 1-particle state density matrix (1DM) or difference density matrix (1DDM). The 1DM is defined in analogy to eqn (2) as

5 *D*^ff^_*pq*_ = 〈*Ψ*_f_|*p*^†^*q*|*Ψ*_f_〉

Furthermore, the difference density matrix (1DDM) is simply the difference between two state 1DMs

6 **Δ**^if^ = **D**^ff^ − **D**^ii^

Diagonalization of the 1DDM and separation of the eigenvectors according to their signs provide detachment and attachment densities.^35,36^ The sum over all positive or negative eigenvalues of the 1DDM—denoted as the promotion number *p*—gives the total number of electrons rearranged during the excitation process. In principle, *p* could lie between zero and the total number of electrons, but in practice, it usually ranges from 1 to 2. The promotion number appears to be a natural measure for defining a multiply excited character. However, since it is not only affected by the electrons taking part in the primary excitation process but also by secondary orbital relaxation,^35,39^*p* has been considered an unsuitable measure.^14^ More generally, doubly excited states usually experience increased *p* values, but increased *p* values alone are not a sufficient criterion to assign doubly excited character.

As an alternative measure for double excitation character, the excitation number (*η*) was introduced by Barca *et al.*^14^ in the context of the maximum overlap method (MOM). For two single-determinantal wave functions, *Φ*_i_ and *Φ*_f_, *η* is defined as7
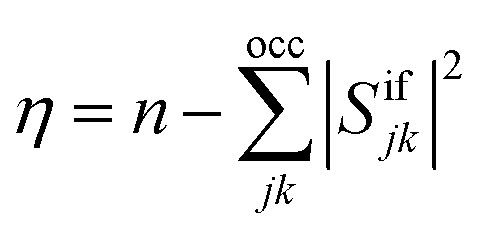
where *S*^if^_*jk*_ is the overlap between the *j*-th occupied orbitals of *Φ*_i_ and the *k*-th occupied orbital of *Φ*_f_; *n* is the total number of electrons in the system. The *η* value ranges from 0 (when *Φ*_i_ is equal to *Φ*_f_) to *n* (when there is no overlap between any orbitals in *Φ*_i_ and *Φ*_f_). A generalization of *η* to arbitrary wave functions is not trivial, and we discuss this issue in some detail in Section S3.† After several attempts, we suggest using the formula

8 *η* = *n*_eff_ − tr(**D**^ii^**D**^ff^)/2

where **D**^ii^ and **D**^ff^ are the spin-traced 1DMs. The value of *n*_eff_ in this equation is defined as

9 *n*_eff_ = max(‖**D**^ii^‖^2^,‖**D**^ff^‖^2^)/2

This expression reduces to eqn (7) for a single-determinantal wave function with doubly occupied spatial orbitals. Furthermore, just like the original expression, it is invariant to a switch between the *initial* and *final* states and vanishes if the *initial* and *final* states are the same. Moreover, we have verified that this expression yields the expected result in model systems and a variety of realistic computations.

Finally, we want to point out that *p* and *η* are both based on the 1DMs, and thus, they analyze shifts in the electron density rather than probing the actual wave functions. Crucially, if the initial and final state should have the same 1DMs, then *p* and *η* would both vanish, formally classifying the state as a “zero-electron transition”. This complicates the assignment in cases of strong ground-state correlation. For example, states where the HOMO and LUMO are singly occupied (HOMO^1^LUMO^1^) or states constructed as a linear combination of the configurations with doubly occupied HOMO or doubly occupied LUMO (HOMO^2^−LUMO^2^, HOMO^2^+LUMO^2^), all possess the same 1DMs, and, thus, the *p* and *η* values between them would vanish.

### 2.3 Further descriptors

If the state is predominantly singly excited (*Ω* ≈ 1), then it is meaningful to analyze the 1TDM further and to obtain the natural transition orbitals (NTOs), defined as the singular vectors of the 1TDM.^40,41^ The number of independent NTO pairs necessary to describe the transition, called the NTO participation ratio,^42,43^ is defined as10
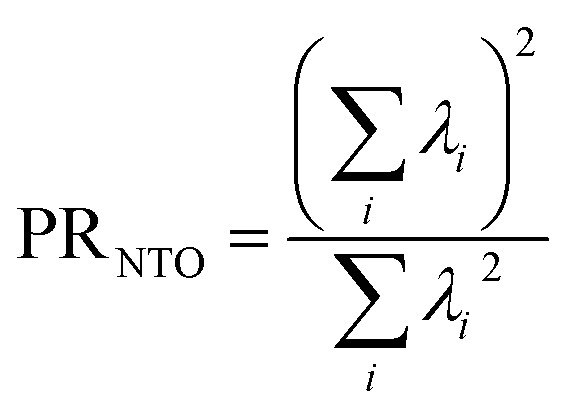
where *λ*_i_ are the weights of the NTO pairs. PR_NTO_ can be used to assess the multiconfigurational character of the transition. A value of 1 means that a single configuration state function can express the excitation; higher values imply that this state has a multiconfigurational nature.

Furthermore, we compute the spin-averaged natural orbitals (NOs), defined as the eigenvectors of the 1DM. Their occupation numbers (*n*_*p*_) range from 0 (unoccupied) to 2 (doubly occupied). It is common to characterize the 1DM *via y*_0_ and *y*_1_, which correspond to the occupation numbers of the lowest unoccupied natural orbitals (LUNO) and LUNO+1, respectively. For example, (*y*_0_, *y*_1_) equals (0,0), (1,0), and (1,1) correspond to a closed shell, pure diradical, and pure tetra-radical characters, respectively.^38,44^ In Section 2.4, we show how these quantities can differentiate between types of doubly excited states. Alternatively, one can also compute the number of effectively unpaired electrons by summing over all NOs of the system. Eqn (12) and (13) show two expressions to obtain the number of unpaired electrons *via* either11
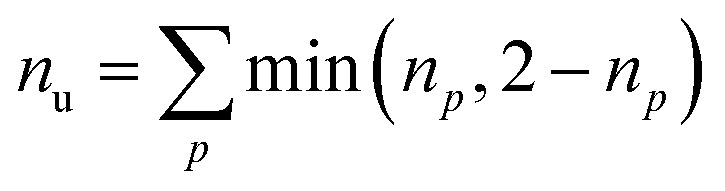
or12
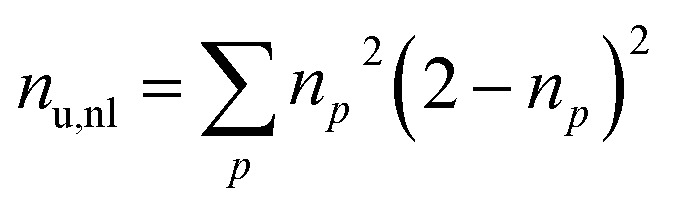
where *n*_u_ includes both static and dynamic correlation, while *n*_u,nl_ suppresses dynamic correlation, thus, focusing on static contributions.^31,37^ In principle, *n*_u_ and *n*_u,nl_ range from zero to the number of electrons. A value of zero represents a closed shell; a value of two a biradical with two open-shell orbitals, four represents a tetra-radical with four open shells, *etc.*

### 2.4 Classification of singly and doubly excited states

**Fig. 2:**
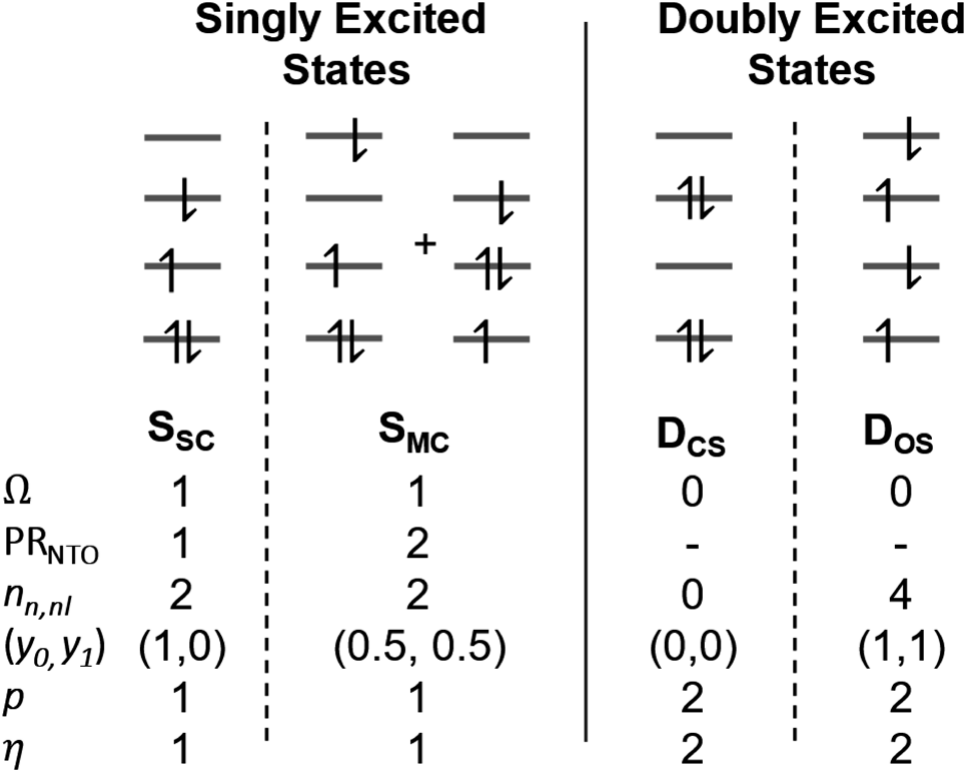
Limiting cases for singly and doubly excited states using a four-orbital four-electron model. Singly excited states distinguish between single configurational (S_SC_) and multiconfigurational (S_MC_) cases; doubly excited states distinguish between the formal closed-shell (D_CS_) and four open-shells (D_OS_) cases.

The descriptors presented above provide a toolbox for a comprehensive description of electronic excitation processes. This section shows how they can be combined to give a well-defined classification scheme of singly and doubly excited states. For this purpose, we discuss the values of the descriptors for four limiting cases: a single configurational (S_SC_) and a multiconfigurational (S_MC_) singly excited state, and a closed-shell (D_CS_) and an open-shell (D_OS_) doubly excited state (Fig. 2). In addition, we will consider the mixed case (D_mix_) as a case with notable doubly excited character not conforming with any of the four limiting cases.

Herein we use *Ω* as the main characteristic to distinguish between singly (*Ω* = 1) and doubly (*Ω* = 0) excited states. Alternatively, *p* and *η* can be used where their values correspond to the number of excited electrons. Note, however, that *p* is also strongly affected by orbital relaxation,^35,39^ and it is unclear how *η* performs in the case of static ground-state correlation.

Within the singly excited states, we distinguish between the single-configurational (S_SC_) and multiconfigurational (S_MC_) limiting cases. In the single-configurational limiting case (S_SC_), the overall excitation can be described as a transition between a single pair of orbitals, *e.g.*, the HOMO → LUMO transition. More generally, we define the S_SC_ case as a state with only a single contributing NTO pair, leading to a value of PR_NTO_ = 1. For the S_SC_ case, (*y*_0_, *y*_1_) is equal to (1, 0) since only one virtual orbital is involved. The multiconfigurational case (S_MC_) is obtained if at least one additional pair of NTOs contributes to the state. In the scheme presented in Fig. 2, S_MC_ is represented by PR_NTO_ = 2 and (*y*_0_, *y*_1_) = (0.5, 0.5). The significance of these differences is discussed in the literature, for example, in the context of excitons and the L_a_/L_b_ states in aromatic molecules.^43,45–48^

Within the doubly excited states, we distinguish between the closed-shell (D_CS_) and open-shell (D_OS_) limiting cases. In the first case, exemplified by a pure HOMO^2^ → LUMO^2^ transition, two electrons are promoted to the same virtual orbital, and the excited state obtains a closed-shell character (D_CS_) similar to the ground state. The second is exemplified by a combined HOMO/HOMO−1 → LUMO/LUMO+1 transition: two electrons are promoted from two different initial orbitals to two different final orbitals, leaving four open-shell orbitals in total. D_CS_ states can only be realized for singlets due to the Pauli principle, whereas D_OS_ states can be singlet, triplet, or quintet. Within the presented scheme, the D_CS_ and D_OS_ states are resolved *via* NO occupations. The D_CS_ limiting case possesses only closed shells and therefore has *n*_u,nl_ = 0 and (*y*_0_, *y*_1_) = (0, 0), in analogy to a closed-shell ground state. Here, the LUMO of the ground state becomes a strongly occupied MO of the excited state. The four open-shell orbitals in the D_OS_ case, on the other hand, are represented by *n*_u,nl_ = 4 and (*y*_0_, *y*_1_) = (1, 1).

The idealized D_CS_ state is single-configurational and behaves like a closed-shell ground state. Such a state would be readily described by a single determinant and would be particularly amenable to the maximum overlap method (MOM).^49^ Furthermore, a CAS(2,2) active space or a single spin flip from a triplet reference would both suffice to describe such a state. On the other hand, a D_OS_ case would always require a more sophisticated treatment, including at least four active orbitals. Similarly, the D_mix_ case requires several correlated orbitals to describe its multiconfigurational character (unless the required nondynamic correlation effects can be captured within the exchange–correlation functional employed). We shall explore these issues below in Section 4.2.

### 2.5 Energies of doubly excited states of monomers and dimers

**Fig. 3:**
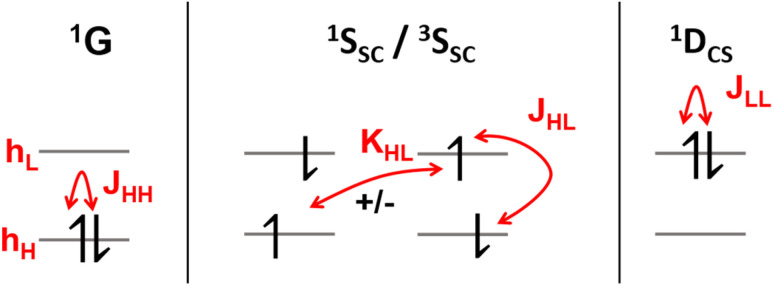
Excited-state diagrams constructed within a two-orbital two-electron model. Energy terms are shown in red: one-electron energies of HOMO (*h*_H_) and LUMO (*h*_L_), the three Coulomb terms (*J*_HH_, *J*_HL_, *J*_LL_), and the exchange term (*K*_HL_).

It is instructive to start by presenting the energies of the various states that can be constructed within a two-orbital two-electron model (TOTEM), as shown in Fig. 3 (see also ref. 8, 16 and 50). For simplicity, we consider transitions from HOMO to LUMO. Four spin-adapted wave functions can be constructed within the TOTEM: the ground state (^1^G) with a doubly occupied HOMO, the single configurational singly excited states (^1^S_SC_/^3^S_SC_) of singlet and triplet multiplicities, and the closed-shell doubly excited state (^1^D_CS_). The relevant energy terms are the one-electron energies of HOMO and LUMO (*h*_H_, *h*_L_), the three Coulomb integrals (*J*_HH_, *J*_HL_, *J*_LL_), and the exchange integral (*K*_HL_). Fig. 3 indicates the different energy terms contributing to the energy. The energy of the ground state (^1^G) is determined by the one-electron energy of the HOMO (*h*_H_) and the Coulomb integral between the two electrons located in the HOMO; the energy of the doubly excited state (^1^D_CS_) is determined in a completely analogous way only that the HOMO is swapped for the LUMO. Determining the energies of the singlet and triplet S_SC_ states is slightly more involved since they are described by two interacting configurations, meaning that an exchange term (*K*_HL_) also comes into play. In summary, the energies of the states are given as

13 *E*(^1^G) = 2*h*_H_ + *J*_HH_

14 *E*(^1^S_SC_) = *h*_H_ + *h*_L_ + *J*_HL_ + *K*_HL_

15 *E*(^3^S_SC_) = *h*_H_ + *h*_L_ + *J*_HL_ − *K*_HL_

16 *E*(^1^D_CS_) = 2*h*_L_ + *J*_LL_

Before continuing, we note that the one-electron energies used above include the kinetic energy, the nucleus-electron attraction and possibly the interaction with any other electrons present (treated in the sense of a frozen core) but do not consider any terms involving the HOMO and LUMO. An alternative and equally valid viewpoint is provided in ref. 8 by using the orbital energies (*ϵ*_H_ = *h*_H_ + *J*_HH_, *ϵ*_L_ = *h*_L_ + 2*J*_HL_ – *K*_HL_) that already include interactions within the HOMO and the LUMO.

Using the TOTEM, we can now examine under what circumstances a closed-shell doubly excited state can be of lower energy than a singly excited state. Solving for *E*(^1^D_CS_) < *E*(^1^S_SC_) with the definitions given above, we obtain

17 *h*_L_ − *h*_H_ < *K*_HL_ + *J*_HL_ − *J*_LL_ ≈ *K*_HL_

where the right-hand side was simplified under the assumption that the *J*_HL_ and *J*_LL_ Coulomb integrals are of similar magnitude.^8^ In other words, the doubly excited state becomes favorable if the exchange repulsion is large compared to the difference in one-electron energies between HOMO and LUMO.

Note, however, that eqn (17) and the assumption that the Coulomb integrals are of similar magnitude would also imply that *E*(^3^S_SC_) < *E*(^1^G), *i.e.* that the triplet lies below the closed shell.^8^*K*_HL_ also couples ^1^G and ^1^D_CS_, meaning that the singlet ground state would obtain multiconfigurational character if the exchange interaction were, indeed, of the same order of magnitude as the difference in one-electron energies. This discussion highlights that a large exchange interaction relatively favors doubly excited states by pushing ^1^S_SC_ up in energy. Nevertheless, it also shows that a simple HOMO^2^ → LUMO^2^ transition cannot be the lowest excited state if the ground state is a closed shell. Indeed, the connection between doubly excited character and static electron correlation in the ground state is emphasized in the literature.^13,14^

Noting that a double excitation from HOMO to LUMO is not feasible for low-energy excited states, we proceed to an alternative type of doubly excited state. This alternative is present in the case of a dimer where two locally excited states can be combined into one doubly excited state of D_OS_ type. Such states can be classified according to the spin-multiplicity of the overall state along with the individual transitions. By combining singlet or triplet states on each monomer and considering all possible spin couplings, one obtains the states ^1^(TT), ^3^(TT),^5^(TT), ^3^(ST),^3^(TS), and ^1^(SS). Here, the ^1^(TT) state is crucial for the singlet fission process, where one high-energy singlet excited state can be converted into two low-energy triplets.^3^ (See also ref. 52 for a discussion of the ^1^(TT) state in the context of spin-exchange internal conversion and ref. 1 for a discussion of intramolecular doubly excited states in singlet fission.) We can use the TOTEM model to evaluate the stability of such a state. If we neglect possible biexciton binding effects, the ^1^(TT) state will be the lowest state of singlet multiplicity if twice the excitation energy of ^3^S_SC_ is lower than the excitation energy of ^1^S_SC_. More specifically,

18 *h*_L_ − *h*_H_ < 3*K*_HL_ + *J*_HH_ − *J*_HL_ ≈ 3*K*_HL_

This condition is certainly easier to satisfy than eqn (17). In a dimer, the exchange repulsion associated with ^1^S_SC_ is avoided, and two exchange integrals are gained due to the two ^3^S_SC_ configurations present.

As discussed below, we found that low-energy D_OS_ type states can be readily constructed in dimers. Conversely, we were unable to find any low-lying D_CS_-type states in a variety of investigated molecules. The D_CS_ states that were indeed found were of σσ* or nπ* character and trivially lay at about twice the energy of the corresponding singly excited state. Importantly, the low-lying A_g_ ππ* states of polyenes and related systems do not fit either the D_OS_ or D_CS_ limitting case. Therefore, we introduce a third class of excited state, D_mix_, which is characterized by appreciable double excitation character (as determined by *Ω* and *η*) but not conforming to either of the limitting cases. Reviewing the TOTEM, we note that it is a simplified model capturing only the D_OS_ and D_CS_ cases but is unable to account for the energies of D_mix_ type states. Indeed, a more involved model combining a triplet-pair state and charge-transfer exciton has been suggested for the latter.^51^

## 3. Computational details

Ground state geometry optimizations and vibrational frequencies of ethylene, butadiene, hexatriene, octatetraene, decapentaene, dodecahexatriene, *s*-tetrazine and the tetracene dimer were obtained at density functional theory (DFT) level with CAM-B3LYP functional,^53^ cc-pVTZ^54^ basis set, and Grimme's D3 dispersion correction.^55^ DFT calculations were done using Gaussian 16 rev a03.^56^ Vertical excitation energies and wave functions obtained at DFT/MRCI level^57^ employed the def2-TZVP^58^ basis set, except for the tetracene dimer where we use def2-SV(P).^58^ In this approach, the CI expansion is built from Kohn–Sham orbitals using the BH-LYP^59^ functional (as implemented in TURBOMOLE 7.5 ^60^) and an effective Hamiltonian. Here, we tested two different parametrizations: the original one proposed by Grimme and Waletzke^57^ and R2018 proposed by Marian *et al.*^61^ As shown in Fig. S1 of the ESI,† R2018 does not reproduce the inversion between states 1^1^B_u_ and 2^1^A_g_ observed in polyenes with the increase of the number of double bonds in the system,^62^ while the original parametrization does.^63^ However, both parametrizations yield similar trends regarding the wave function analysis (Fig. S2†). In this work, we chose to use the original set of parameters. The initial reference space included configurations obtained from single and double excitations of 10 electrons within 10 orbitals.

TDDFT calculations were performed with the BLYP functional^64,65^ and 6-31G** basis set^66^ with Q-Chem 5.3.^67^ Multireference configuration interaction with single and double excitations (MR-CISD) were carried out using COLUMBUS 7.0.^68–70^ A complete active reference space (CAS) including all π and π* orbitals was used for polyenes up to four double bonds; polyenes with five and six double bonds were restricted to a CAS(8,8) due to computational cost. A complete active space self-consistent field (CASSCF) with these active spaces was used to construct the orbitals using state-averaging over the first two A_g_ and the first B_u_ states. MRCI energies are reported using the Pople extensivity correction (+P); 1s orbitals of all carbon atoms were frozen.^71^ Vertical excitation energies were also computed at the third-order algebraic diagrammatic construction method (ADC(3)) for the polarization propagator^20,72^ level with the resolution-of-identity approximation and def2-SV(P) basis set, as implemented in Q-Chem.

In ADC(3) calculations, the wave function analysis library (libwfa)^31,32^ was used to obtain *Ω*-values, participation ratio of the natural transition orbitals (PR_NTO_), occupation of natural orbitals (*y*_0_ and *y*_1_), number of unpaired electrons (*n*_u,nl_), and promotion number (*p*). *η* at ADC(3) and all descriptors at DFT/MRCI and MRCI levels were obtained externally with TheoDORE^73^ analysis package using a pre-release of version 3.0.

To investigate the cycloaddition of ethylene, we performed a relaxed scan keeping the linear combination of C1–C3 and C2–C4 (Fig. 1) at fixed distances. Ground state geometry optimizations were done at DFT level using B3LYP/cc-pVTZ. Excited-state energies and wave functions were obtained at MRCI+P(8,8)/cc-pVDZ level. All calculations considered *D*_2h_ symmetry. CAS(8,8) wave functions with 6 states in the average (four A_g_ and two B_u_) were used as references. MRCI calculations considered 4 frozen core orbitals (belonging to a_g_, b_3u_, b_2u_, and b_1g_ representations), and 1 orbital in the active space for each of the 8 irreducible representations of the *D*_2h_ point group.

## 4. Results and discussion

### 4.1 Formaldehyde dimer

First, we discuss the ten lowest excited states of the formaldehyde dimer at 5 Å intermolecular distance calculated at the ADC(3) level (Table 1). The first six excited states are excitonic combinations of the ^3^nπ*, ^1^nπ*, and ^3^ππ* states on the monomers. They are predominantly singly excited, as seen by their *Ω* values near or above 0.9 and *η* values close to 1.0. Their PR_NTO_ values close to 2 (along with appropriate *n*_u,nl_, *y*_0_, and *y*_1_ values) allow us to classify them as multiconfigurational (S_MC_) states, according to Fig. 2. The multiconfigurational nature of excitonic states delocalized between two interacting chromophores has been discussed in detail elsewhere,^43,45^ and we shall proceed with the remaining states here. The final four states are unambiguously characterized as doubly excited due to their *Ω* values of exactly 0.000, along with *η* values near 2. More specifically, we find that these four states comply with the definition of an open-shell doubly excited state (D_OS_) in Fig. 2, and all relevant descriptors are within 0.05 of their idealized values. This discussion highlights that D_OS_ states are indeed readily constructed in realistic systems and using a high-level method.

**Table 1:** Excitation energy (Δ*E* in eV), oscillator strength (*f*), and wave function descriptors of the lowest excited singlet and triplet states of the formaldehyde dimer at 5 Å intermolecular separation at ADC(3) level

State	Chars	Δ*E* (eV)	*f*	*Ω*	PR_NTO_	*n*_u,nl_	*y*_0_	*y*_1_	*p*	*η*
1^3^A_2_	^3^nπ*	3.67	—	0.909	2.02	2.46	0.543	0.525	1.15	1.01
1^3^B_2_	^3^nπ*	3.67	—	0.909	2.02	2.46	0.543	0.526	1.15	1.01
1^1^A_2_	^1^nπ*	4.12	0.000	0.882	2.01	2.51	0.556	0.537	1.15	1.03
1^1^B_2_	^1^nπ*	4.12	0.000	0.882	2.01	2.51	0.555	0.537	1.15	1.03
1^3^B_1_	^3^ππ*	6.08	—	0.945	2.09	2.44	0.526	0.510	1.16	0.95
1^3^A_1_	^3^ππ*	6.08	—	0.945	2.09	2.44	0.523	0.513	1.16	0.95
2^1^A_1_	nπ*:^1^(TT)	7.68	0.000	0.000	—	4.05	0.973	0.972	2.00	1.95
2^3^B_1_	nπ*:^3^(TT)	7.68	—	0.000	—	4.05	0.973	0.972	2.00	1.95
3^3^B_1_	nπ*:^3^(ST)	8.53	—	0.000	—	4.05	0.972	0.971	2.00	1.95
2^3^A_1_	nπ*:^3^(ST)	8.53	—	0.000	—	4.05	0.972	0.972	2.00	1.95

We find that the excitation energy of the 2^1^A_1_ state (7.68 eV) is about twice the excitation energy of the singly excited ^3^nπ* state (3.67 eV), and we, therefore, assign it as the corresponding ^1^(TT) state. Note, however, that the agreement is not perfect, and the ^1^(TT) state lies about 0.35 eV higher than expected using the monomer energies. In principle, such a difference could derive from (bi)excitonic interaction effects. However, these are probably negligible at 5 Å, as the states come in pairs of the same energy. Conversely, we ascribe the difference to a lack of internal consistency within ADC(3) in terms of describing singly and doubly excited states at exactly the same level (see Section S6.1 in the ESI†). The next doubly excited state is of triplet multiplicity and almost degenerate with ^1^(TT); it is assigned as the ^3^(TT) state. The final two states shown in Table 1 combine singlet and triplet monomer excitations to form a ^3^(ST) state and are, again, almost degenerate. Their energy (8.53 eV) is significantly higher than the combined ^3^nπ* and ^1^nπ* energies (7.79 eV), which we again attribute to a lack of complete internal consistency within ADC(3).

### 4.2 Butadiene and larger polyenes

The photophysics of polyenes is usually discussed in terms of two important states close in energy: the 1^1^B_u_ state dominated by the HOMO → LUMO transition and the 2^1^A_g_ state with at least partial admixture of the doubly excited HOMO^2^ → LUMO^2^ configuration.^9,10^ Within the following, we study these states in different polyenes using a variety of computational methods. We emphasize that our primary goal is not to obtain an accurate energy gap value between these states but rather to elucidate the nature of the 2^1^A_g_ state.

The characterization and excited state ordering of polyenes is still a subject of discussion in the literature. Experimentally, 2^1^A_g_ becomes the lowest excited state for polyenes with more than four double bonds.^8,74^ Computationally, the energy gap and order of states are strongly method-dependent.^20,63,75,76^ While MS-CASPT2 calculations correctly predict the state inversion, CC3 predicts that 1^1^B_u_ remains above 2^1^A_g_, although both methods deliver a small energy gap between those states.^63^ ADC(2)-x and ADC(3) always predict 2^1^A_g_ state as the lowest excited state, while ADC(2)-s predicts that to be the 1^1^B_u_ state for polyenes up to four double bonds.^20^ Benchmark studies show that the gap between 2^1^A_g_ and 1^1^B_u_ in polyenes is reproduced correctly by DFT/MRCI, although the excitation energies are lower than the best theoretical estimate.^61,63,75^ Due to these discrepancies, here we use four different computational methods to study polyene excited states: ADC(3), MRCI, DFT/MRCI, and TDDFT/BLYP. For DFT/MRCI we use the original parameterisation because it reproduces the inversion between 1^1^B_u_ and 2^1^A_g_ expected in larger polyenes; see ESI Section S4.†

In particular, butadiene has become a paradigmatic case for discussing doubly excited states.^8,13,14^ Thus, we first focus on this molecule using the ADC(3) method. To obtain a comprehensive picture, we look at several density descriptors (following Fig. 2) along with the percentage of single excitations (%*T*_1_) within ADC(3). The results are presented in Table 2. We start the discussion with the 1^1^B_u_ state, which at this level of theory is the second excited state lying at 6.72 eV. *Ω* and *η* values close to 1 unambiguously assign the state as being singly excited. Furthermore, PR_NTO_ approximately 1, along with (*y*_0_, *y*_1_) = (0.965, 0.039), allows classifying it as an S_SC_ single configurational state close to the idealized values presented in Fig. 2.

**Table 2:** Excitation energy (Δ*E* in eV), oscillator strength (*f*), and wave function descriptors of the lowest excited singlet states of butadiene computed at the ADC(3) level

State	Δ*E* (eV)	*f*	*Ω*	PR_NTO_	*n*_u,nl_	*y*_0_	*y*_1_	*p*	*η*	%*T*_1_
1^1^A_g_	—	—	—	—	0.186	0.071	0.053	—	—	—
2^1^A_g_	6.02	0.000	0.305	1.959	2.430	0.890	0.253	1.672	1.537	31.3
1^1^B_u_	6.72	1.739	0.904	1.095	2.073	0.965	0.039	0.998	0.899	93.5

The assignment of 2^1^A_g_ of butadiene, which lies at 6.02 eV, is more involved. An *Ω*-value of 0.305, a %*T*_1_ value of 31.3%, and an excitation number (*η*) of 1.537 indicate a partially doubly excited character, which is also supported by a promotion number (*p*) of 1.672. However, the descriptors are notably different from the idealized case of a doubly excited state (*Ω* = 0, *η* = *p* = 2) shown in Fig. 2. This implies that the admixture of singly excited configurations plays an important role. The dominant contribution to the 2^1^A_g_ state is the HOMO^2^ → LUMO^2^ transition, with a weight of 42%. The HOMO−1 → LUMO (18%) and HOMO → LUMO+1 (13%) transitions come next, followed by many doubly excited configurations, all involving the HOMO−1, HOMO, LUMO, and LUMO+1. Thus, this state is strongly multiconfigurational, with notably different characteristics to the idealized D_CS_ case. The NO-based characteristics reflect this divergence particularly well. For the idealized D_CS_ case, *n*_u,nl_, *y*_0_, and *y*_1_ are all zero. However, for the 2^1^A_g_ state of butadiene, these values are *n*_u,nl_ = 2.430, and (*y*_0_, *y*_1_) = (0.890, 0.253), which are between the limiting cases shown in Fig. 2. For these reasons, we classify this state as D_mix_.

As a next step, we investigate the dependence of the presented results on the electronic structure method and the size of the molecule. For this reason, we computed excitation energies and 1(T)DM descriptors at MRCI, DFT/MRCI, and ADC(3) levels for all-*trans*-butadiene, all-*trans*-hexatriene, all-*trans*-octatetraene, all-*trans*-decapentaene, and all-*trans*-dodecahexene (*N* = 2, 3, 4, 5, 6, respectively, where *N* is the number of double bonds in the system).

**Fig. 4:**
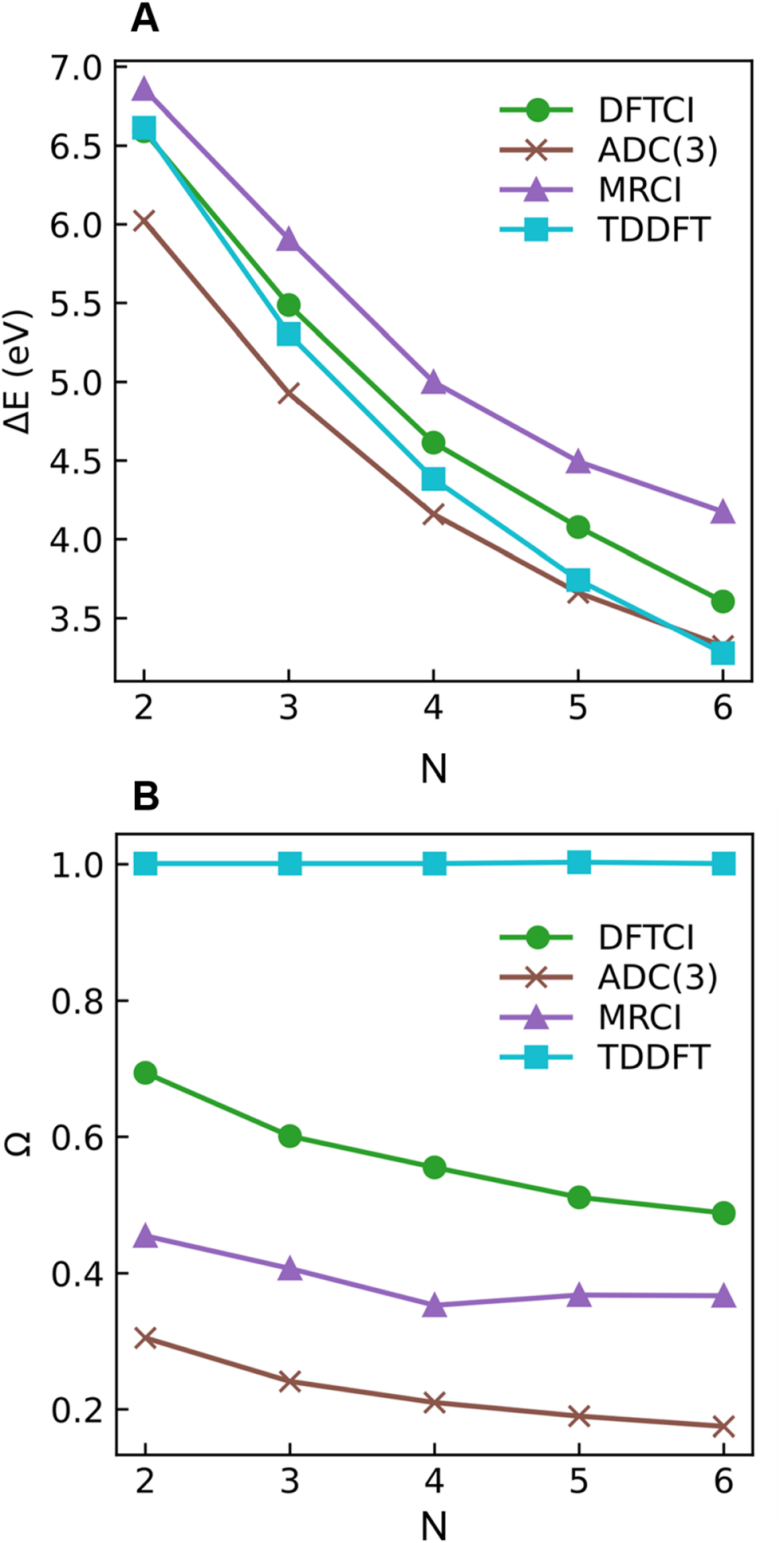
Excitation energies Δ*E* (A) and 1TDM norm *Ω* (B) of the 2^1^Ag state of different polyenes plotted against the number of double bonds (*N*) calculated at ADC(3), DFT/MRCI (dubbed DFTCI in the insert), MRCI, and TDDFT/BLYP levels.

Starting with the excitation energies (Fig. 4A), we find a substantial decrease with increasing *N* for all methods. However, there is a notable difference in the 2^1^A_g_ excitation energies according to the trend ADC(3) < TDDFT < DFT/MRCI< MRCI+P, spanning a range of up to ≈1 eV. Concerning the *Ω* values presented in Fig. 4B, all methods aside from TDDFT find substantial double excitation character (*Ω* < 0.65) for all the molecules. The *Ω* values generally decrease with increasing *N*. The only exception is for the largest MRCI computations, which may be affected by size-extensivity problems. Aside from the general trends, there is also a quite notable difference in the obtained *Ω* values. ADC(3) indicates strong double-excitation character (*Ω* < 0.30 for *N* > 2); MRCI delivers intermediate values (0.35 < *Ω* < 0.5); DFT/MRCI predominantly indicates single excitation character (*Ω* > 0.5) albeit with notable admixtures of double excitations. TDDFT/BLYP, on the other hand, always predicts a singly excited character (*Ω* ≈ 1). The low *Ω* values for 2^1^A_g_ contrast with the high *Ω* values for 1^1^B_u_ (Fig. S9†), which are consistently above 0.90 for DFT/MRCI and TDDFT and above 0.85 for ADC(3) and MRCI. Thus, the *Ω* values clearly distinguish between the wave functions of the 2^1^A_g_ and 1^1^B_u_ states, assigning a partial doubly excited character to 2^1^A_g_.

As outlined in Section 2.1, *Ω* has a clear physical meaning by acting as an effective proportionality factor specifying how strongly the states are coupled *via* one-electron operators. Changes in *Ω* are related to changes in physically observable transition properties. In the present case, the transition dipole moments of the A_g_ states vanish for symmetry reasons, but the differences should show up *via* enhanced transition quadrupole moments or angular momenta when computed with methods that produce different *Ω* values. However, this discussion is left to future work.

**Fig. 5:**
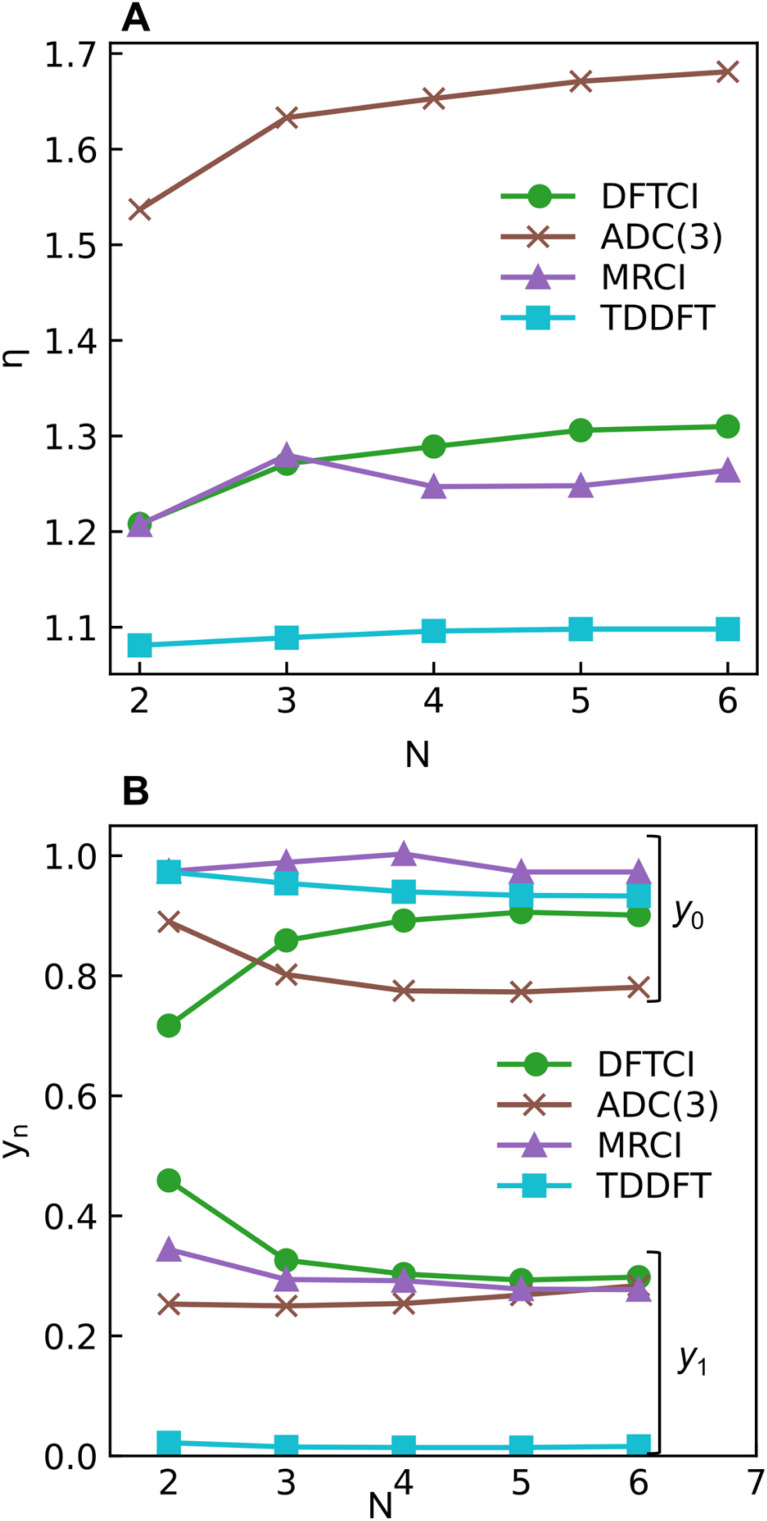
Excitation number *η* (A) and occupations (B) of the lowest unoccupied natural orbital (LUNO, *y*_0_) and LUNO+1 (*y*_1_) of 2^1^A_g_ state of different polyenes plotted against the number of double bonds (N) calculated at ADC(3), DFT/MRCI (dubbed DFTCI in the insert), MRCI, and TDDFT/BLYP levels.

The excitation numbers (Fig. 5A) agree with the *Ω*-values in the sense that they always attribute at least partial doubly excited character to the 2^1^A_g_ state (*η* > 1.2). Interestingly, the MRCI and DFT/MRCI values are both fairly low, with *η* ≤ 1.3, whereas ADC(3) provides significantly enhanced doubly excited character (*η* > 1.5). One can understand this discrepancy by noticing that these descriptors are differently affected by ground-state correlation (see Section 2.2). Generally speaking, it is not clear how the excitation number, initially developed for comparing single determinantal wave functions, should be interpreted in the case of multiconfigurational states. Nonetheless, it is interesting to discuss the excitation numbers of the 1^1^B_u_ excited states (Fig. S10†). For MRCI, these are always below 0.85; for DFT/MRCI and ADC(3), values below 0.95 are obtained. Thus, a clear differentiation between the singly excited 1^1^B_u_ and doubly excited 1^1^A_g_ states is also present when the excitation numbers are considered. Finally, the *y*_0_ values (Fig. 5B) are close to 1 for all methods, whereas the *y*_1_ values are smaller than 0.5. Note that these *y*_0_/*y*_1_ values are inconsistent with any limiting cases presented in Fig. 2. Conversely, they illustrate the multiconfigurational and partial singly and doubly excited nature of the states, in line with a D_mix_ character. This is, again, markedly different from the singly excited 1^1^B_u_ states (Fig. S13†), which for all methods aside from TDDFT, exhibit the idealized values (*y*_0_ ≈ 1, *y*_1_ ≈ 0) expected for S_SC_ states.

In summary, we find at least a partial double excitation character, classified as D_mix_, in the 2^1^A_g_ state of all polyenes investigated, revealed by all methods (except TDDFT) and descriptors in agreement with much of the previous literature.^8–10^ Nevertheless, two contradicting viewpoints argue against the double excitation character of butadiene in the literature, which we discuss next.

Shu and Truhlar^13^ have presented butadiene computations at various computational levels to understand the differences between the 2^1^A_g_ and 1^1^B_u_ states and learn which computational methods are suitable for their description. Crucially, they argued that doubly excited states could not be understood separately, but the multireference character of the ground state promotes the contribution of doubly excited configurations in low-lying excited states. This assessment agrees with the data presented in Table 2 and the discussion in Section 2.5. Nonetheless, we emphasise that the unique properties of polyenes cannot be understood by considering the ground state alone. Otherwise, we would observe similar amounts of double excitations in both 2^1^A_g_ and 1^1^B_u_. In contrast, the 2^1^A_g_ and 1^1^B_u_ states possess distinct characters: only 2^1^A_g_ obtains double excitations, while 1^1^B_u_ retains the singly excited (S_SC_) character. Furthermore, Shu and Truhlar have advocated using local functionals to describe the excited states of polyenes.^13^ They argued that local functionals, such as BLYP, revPBE and M06-L, perform well since they minimize the static correlation error included by the Hartree–Fock exchange in nonlocal functionals.^13,77^ Considering only the energies, one finds that TDDFT/BLYP does indeed produce results comparable to the wave-function-based methods (Fig. 4A). However, special care has to be taken due to the differences in *Ω*-values (Fig. 4B), which would predict significantly altered transition properties.

Subsequently, Barca *et al.*^14^ performed DFT/MOM computations on butadiene and analysed them with the help of their original excitation number (*η*) definition, as shown in eqn (7). A value of *η* = 1.022 was obtained for 2^1^A_g_ indicating almost perfect singly excited character. The challenge in interpreting these results is that the correlated computations describe the 2^1^A_g_ state as a mixture of the HOMO−1/LUMO, HOMO/LUMO+1, and HOMO^2^/LUMO^2^ configurations. However, the DFT/MOM method produces only a single open-shell Slater determinant. Thus, it is doubtful whether the MOM method provides a realistic description of this intrinsically multiconfigurational state. Conversely, extending the excitation number to the multiconfigurational case (Fig. 5A) highlights the doubly excited character. Barca *et al.* argue that it is not clear *a priori* whether the doubly substituted determinants in CI wave functions account for electron excitation, electron correlation, or orbital relaxation, not allowing an unambiguous assignment.^14^ It is precisely for this reason that we have chosen a rigorously defined set of density-matrix-based descriptors to avoid such ambiguities. All the proposed descriptors are invariant to the orbital representation employed. This choice consistently shows the admixture of doubly excited characters in the 2^1^A_g_ state of butadiene. It is important to point out that this contrasts with both Barca *et al.*'s assignment as a singly excited state, and Shu and Truhlar's argument that the apparent doubly excited character is due to correlation at the ground state.

### 4.3 Cycloaddition of ethylene

**Fig. 6:**
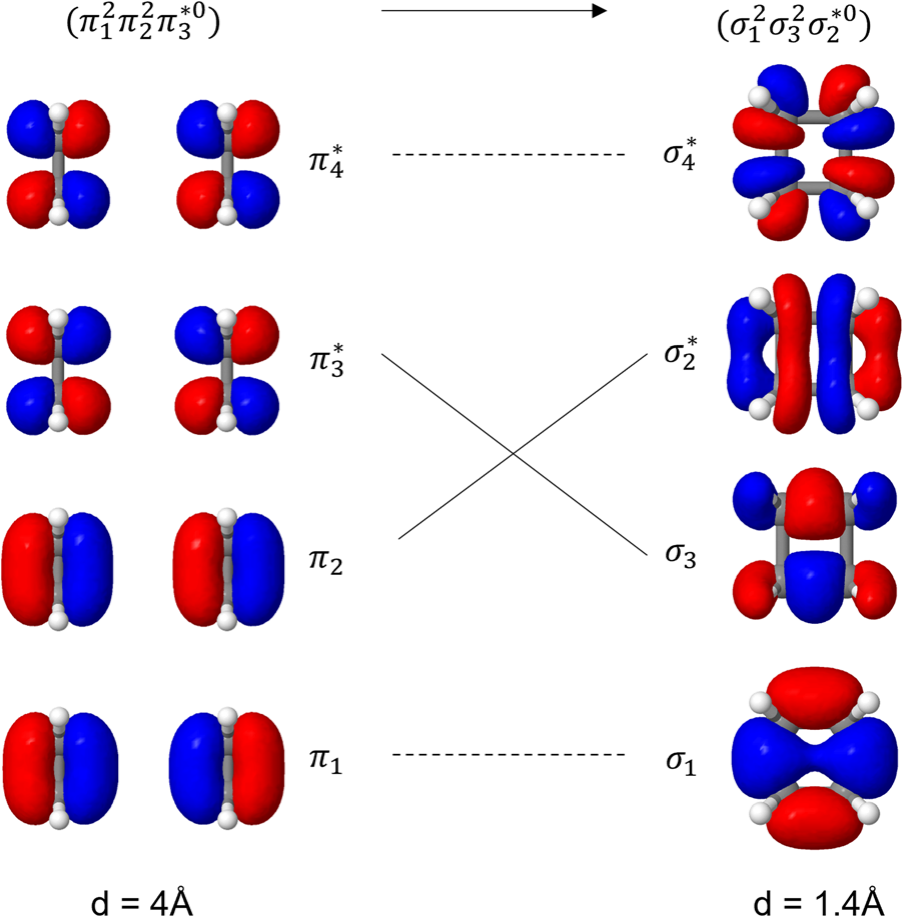
Orbital correlation diagram for two ethylene molecules separated by 4 Å (left) and 1.4 Å (right).

Unable to locate the D_CS_ case in polyenes, we proceed to a third model, the cycloaddition of ethylene. The dimerization of ethylene to cyclobutane is a typical example illustrating the changes in the wave function character along a chemical reaction. According to the Woodward–Hoffmann rules, the [2 + 2] cycloaddition of ethylene is thermally forbidden due to a change in the ground state electronic configuration, yet it is photochemically allowed.^78,79^ The reaction pathway can be explained by the four frontier orbitals shown in Fig. 6. The left represents the case of two isolated ethylene molecules where the frontier orbitals are of π and π* character. As they get closer, the spatial overlap between the orbitals increases until they eventually form the σ and σ* orbitals of cyclobutane shown on the right. Crucially, the HOMO (π_2_) of the isolated ethylene molecules corresponds to the LUMO 
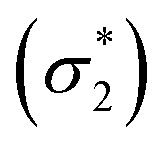
 of cyclobutane and *vice versa*. Thus, the dimerization requires a change in electronic configuration from 
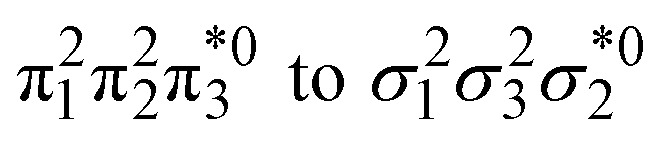
. This process is thermochemically forbidden but can be facilitated *via* a doubly excited state. In this section, we assess the involvement of this doubly excited state, focusing on how its character changes along the reaction coordinate.

The ethylene dimerization is a practical model illustrating the transition from the D_OS_ to D_CS_ limits. The closed- or open-shell character is determined by an interplay between LUMO and LUMO+1 energies and the exchange integral, as outlined in Section 2.5. While the exchange term favors D_OS_, D_CS_ is favored if LUMO and LUMO+1 are farther apart in energy. At large separations, HOMO (π_2_) and HOMO-1 (π_1_), as well as LUMO 
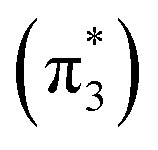
 and LUMO+1 
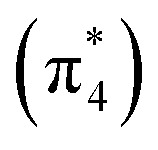
, are pairwise degenerate. Thus, exchange dominates, and the two electrons are promoted to different unoccupied orbitals in a ^1^(TT) type state with a 
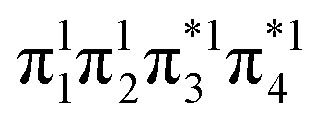
 configuration. As the distance decreases, the overlap between the ethylene orbitals increases, becoming non-degenerate. Then, the LUMO+1 becomes inaccessible, and a D_CS_ state arises. Below, we evaluate the validity of this model using *ab initio* computations.

**Fig. 7:**
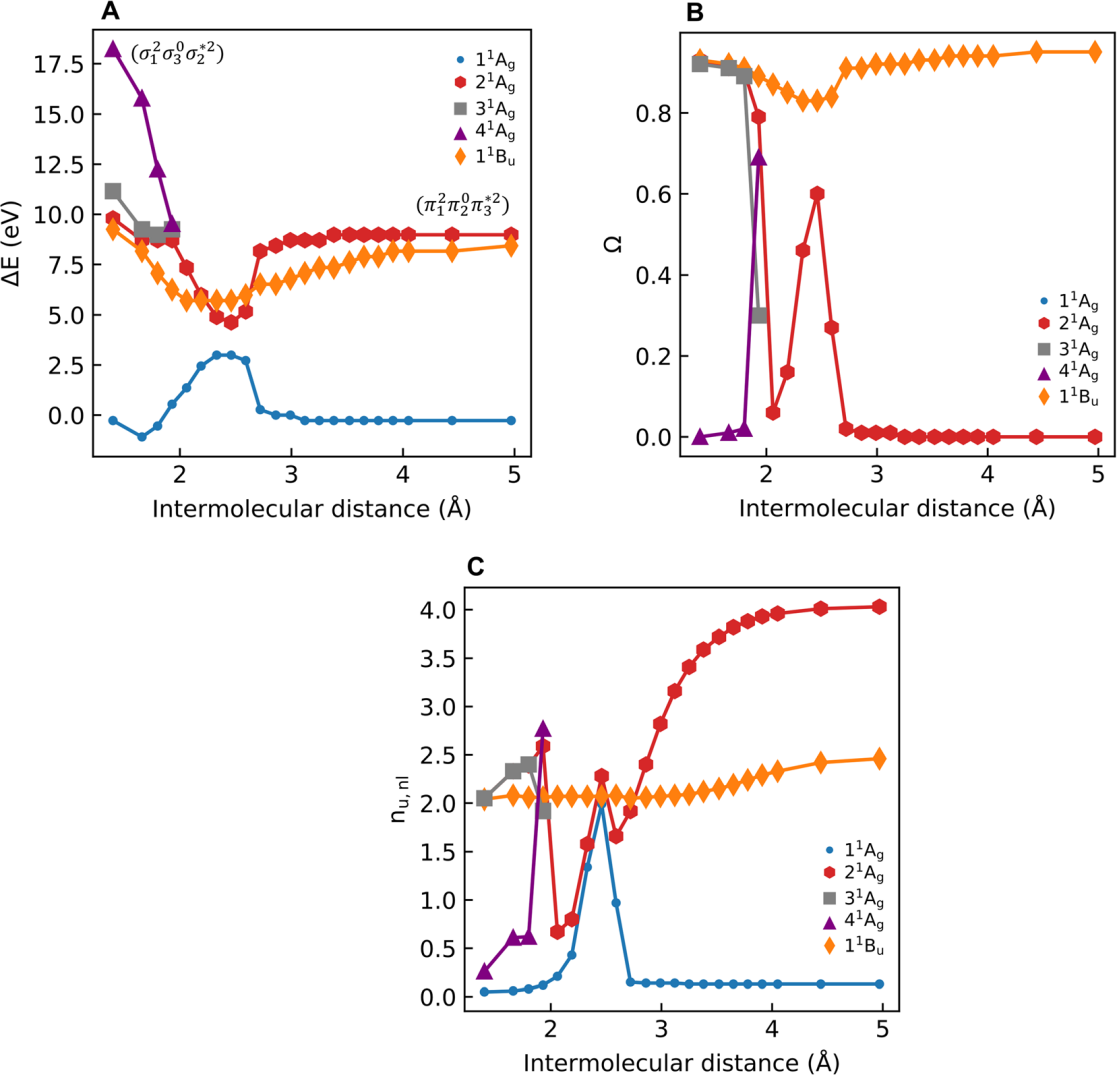
Relative energies in eV (A), 1TDM norms *Ω* (B), and number of unpaired electrons (*n*_u,nl_; C) of selected singlet states of the ethylene dimer/cyclobutane system plotted against the intermolecular separation.

Generally speaking, three states are relevant in the dimerization process, 1^1^A_g_, 2^1^A_g_, and 1^1^B_u_. Here, the closed shell and doubly excited states—that is 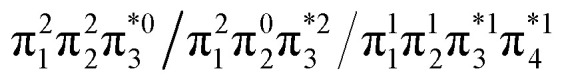 for the dimer and 
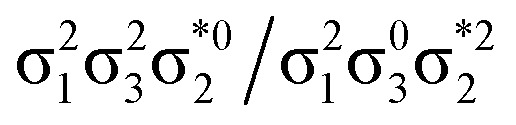
 for cyclobutane—are always of A_g_ symmetry. The singly excited states 
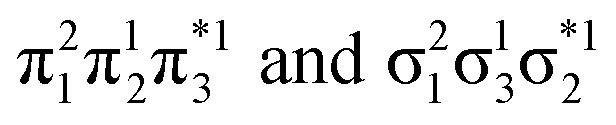
 are always B_u_. Fig. 7A presents the MRCI+P energies of these states computed along a relaxed scan. The right side shows the case of isolated ethylene molecules, whereas the left side represents the formation of cyclobutane. Starting with the 1^1^A_g_ curve, we find that the dimerization is energetically favorable, but a substantial energy barrier of over 2.5 eV is encountered, making the reaction unfeasible in the ground state, as mentioned. Considering the excited states, the singly excited state (1^1^B_u_) is generally below any of the A_g_ states, except in the avoided crossing region, where 2^1^A_g_ becomes lower in energy. The excited states are fairly flat toward the right, whereas a steep increase of the doubly excited state is seen toward the left once cyclobutane is formed. Indeed, the doubly excited state of interest becomes 4^1^A_g_ and reaches an adiabatic energy of 18.5 eV. Fig. 7A suggests a clear mechanism for the photochemical reaction: a photon is initially absorbed by 1^1^B_u_. Subsequently, the two molecules are attracted, forming an excimer. Near the minimum of the excimer, a crossing with 2^1^A_g_ is encountered. Furthermore, 2^1^A_g_ finally relaxes to the 1^1^A_g_ ground state, forming cyclobutane.

To characterize the amount of double excitation character involved, we use the *Ω* descriptor, as presented in Fig. 7B. The *Ω* value for 1^1^B_u_ is close to 1 throughout the energy profile, highlighting the singly excited nature of this state. Conversely, the *Ω* value for 2^1^A_g_ is close to zero on the right side, highlighting that this state is doubly excited in the limit of the separated dimer. On the left side, the doubly excited character is transferred to the 4^1^A_g_ state, as seen by its *Ω* value close to zero. A large spike in 2^1^A_g_ is observed during the first avoided crossing around 2.5 Å. At this point, the 1^1^A_g_ and 2^1^A_g_ states become multiconfigurational, and a clear definition of doubly excited character becomes more challenging. Note that there is an additional spike in the region around 2.0 Å. It is related to a second avoided crossing involving 2^1^A_g_, 3^1^A_g_, and 4^1^A_g_.

The singly and doubly excited nature of the 1^1^B_u_ and 2^1^A_g_ states for reactant and product can also be represented by the *η* and *p* values (Fig. S16 and S17†). Toward the left and right, they represent the singly and doubly excited character, similar to *Ω* values. Interestingly, both *η* and *p* tend towards zero for 1^1^B_u_ and 2^1^A_g_ at the avoided crossing around 2.5 Å, which can be understood following the discussion in the last paragraph of Section 2.2. The 
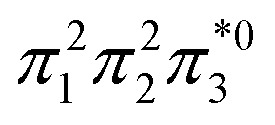
 and 
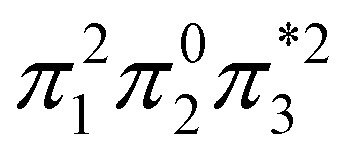
 configurations mix and, as a consequence, all states have the same natural orbitals (with singly occupied π_2_ and 
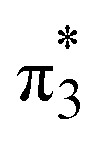
 orbitals) and, hence, the same density matrix.

Having verified the overall amount of doubly excited character, we now use *n*_u,nl_ to obtain a more detailed classification of the states (see Fig. 7C). As expected, *n*_u,nl_ is close to two for the singly excited 1^1^B_u_ state and, aside from the avoided crossing, near zero for the closed-shell 1^1^A_g_ state. For the doubly excited 2^1^A_g_ state, we find that its *n*_u,nl_ value is close to 4 for large intermolecular separations, representing the idealized D_OS_ (^1^TT, that is, 
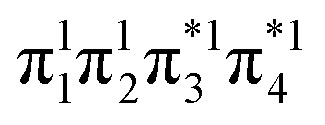
) case. The value steadily decreases as the molecules move together (aside from the avoided crossings). Below the second avoided crossing, the doubly excited state becomes 4^1^A_g_; its *n*_u,nl_ value further decreases until reaching a value of 0.26 for the last point probed. This low *n*_u,nl_ value, along with the individual (*y*_0_, *y*_1_) = (0.16, 0.08) values shown in Fig. S18–S20,† highlights that the state at this geometry does, indeed, closely conform with the idealized D_CS_ state hypothesized in Fig. 2. Note, however, that this doubly excited state occurs at an extremely high vertical excitation energy of 18.5 eV. Indeed, its vertical excitation energy is about twice as high as the singly excited 1^1^B_u_ state. Thus, in line with the above discussion (Section 2.5), we can state that a D_CS_-type state can only occur at energies significantly higher than the lowest singly excited state.

### 4.4 Further molecules

**Fig. 8:**
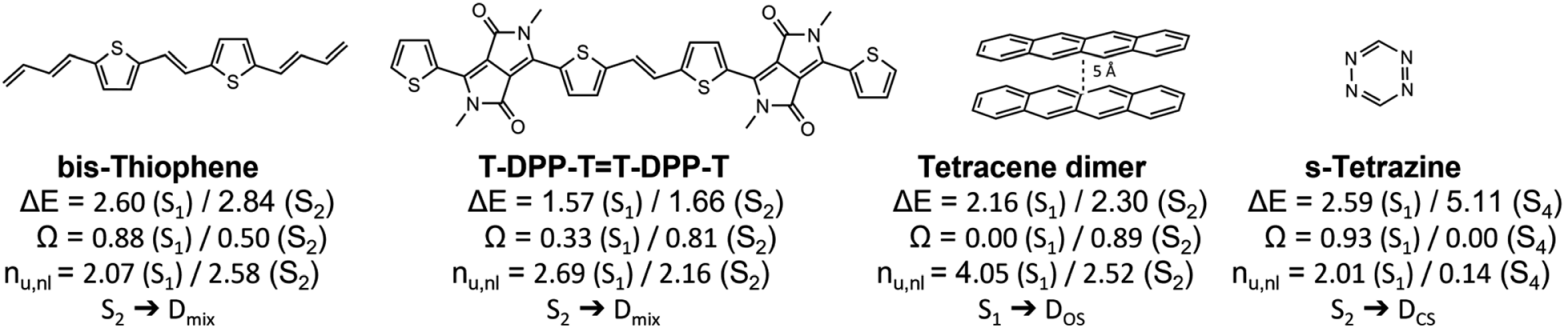
Analysis of the lowest singly and doubly excited singlet states of various molecular systems: excitation energies (Δ*E* in eV), squared 1TDM norms (*Ω*) and numbers of unpaired electrons (*n*_u,nl_).

Having outlined the different archetypes of doubly excited character in some detail above, we applied the proposed scheme to larger and more complex molecular systems. The selected molecules and a summary of the results are presented in Fig. 8, and more data is presented in the ESI.† We start with a diketopyrrolopyrrole derivative^76^ used in optoelectronics,^80,81^ OLEDs^82^ and as singlet-fission chromophores^83,84^ and a derivative of its bis-thiophene building block (Fig. 8). A more extended set of derivatives is given in Table S4.† As pointed out previously,^76^ these molecules have an extended polyene backbone with additional functional groups. The lowest doubly excited singlet states (of A_g_ symmetry) in these molecules lie at similar energies as the lowest singly excited B_u_ singlet states. In both cases shown and all examples in Table S4,† the A_g_ states present the clear signature of the D_mix_ case (0.3 < *Ω* < 0.6, 2.5 < *n*_u,nl_ < 2.8). This analysis highlights that the D_mix_ case applies to a variety of molecules and is an important model to understand electronic excitations.

Other interesting examples are the tetracene dimer and *s*-tetrazine. The tetracene dimer at 5 Å intermolecular separation is chosen as a more realistic illustration of dimer excited states relevant to singlet fission. Its lowest singlet excited state at 2.16 eV is a doubly excited ππ* state delocalized over both molecules. With values of *Ω* = 0.00 and *n*_u,nl_ = 4.05 it almost perfectly aligns with the D_OS_ case highlighting the relevance of this case for general dimer excited states. Finally, we investigate *s*-tetrazine. This molecule possesses a doubly excited nπ* state at 5.11 eV. With values of *Ω* = 0.00 and *n*_u,nl_ = 0.14 it is a close match to the D_CS_ case. Its energy is about twice the energy of S_1_, the singly excited nπ* state. Thus, in agreement with the previous discussions, we find that D_CS_ type states are only found well above the first singly excited state.

## 5. Conclusions

This work presents a rigorous and transferable classification scheme for doubly excited states. We propose to define a doubly (or higher) excited state as a state that cannot be coupled to the ground state with any conceivable one-electron operator. This physically meaningful definition can be readily evaluated numerically using the 1TDM norm.

Within the manifold of doubly excited states, we define three cases: the closed-shell (D_CS_) case, where two electrons are promoted together from one orbital to another; the open-shell case (D_OS_), where the excitations occur between two independent orbital pairs; and the multiconfigurational mixed case (D_mix_) possessing only partial doubly excited character and conforming with neither of the above definitions. The underlying energetics are presented, highlighting that the D_OS_ limiting case can occur as a low-lying excited state in realistic computations. Conversely, the pure D_CS_ case is expected at significantly higher energies, and only D_mix_ is a viable model for low-lying intramolecular doubly excited states. The differentiation between D_OS_, D_CS_, and D_mix_ in practical calculations using natural orbital occupations and other readily available density matrix descriptors is outlined. We find that the assignment of the D_OS_ and D_CS_ cases is generally clear and unambiguous. By contrast, D_mix_ type states are sometimes discussed quite controversially in the literature. Other authors^13,14^ have refrained from using the term “doubly excited” for D_mix_ type states reserving it for the D_OS_ and D_CS_ cases. Ultimately, this is a question of terminology. Nonetheless, this study shows that D_mix_ states possess unique wavefunction properties that are clearly differentiated from traditional singly excited states and we, therefore, suggest labelling them as states with partial doubly excited character.

Several practical examples are presented to study the occurrence of different types of doubly excited states and their description with different computational methods. First, we highlight that D_OS_-type states can be readily constructed in dimers where they occur as different combinations of the monomer singlet and triplet states, such as ^1^(TT), ^3^(TT), ^3^(ST). Computations of the formaldehyde dimer at the ADC(3) level are presented, illustrating that even at this highly correlated level, one obtains 1(T)DM descriptors close to the idealized results.

We proceed to butadiene and larger polyenes to present results on their controversially discussed 2^1^A_g_ excited states. Computations at the ADC(3), *ab initio* MRCI, and DFT/MRCI levels all agree that admixture of doubly excited character *via* the HOMO^2^ → LUMO^2^ transition plays an important role in the 2^1^A_g_ excited states of these molecules. At the same time, the description is never close to a D_CS_ limiting case, but multiconfigurational character and admixture of singly excited configurations play a significant role in line with the D_mix_ case.

Unable to locate the D_CS_ case in polyenes, we proceed to a third model, the [2 + 2] cycloaddition of ethylene. At large intermolecular separations, a doubly excited D_OS_ (^1^TT, 
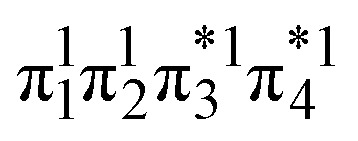
) state is found. Upon dimerization, this state converts into a near-perfect D_CS_
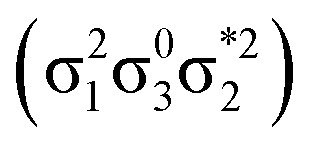
 state that is strongly dominated by the HOMO^2^ → LUMO^2^ transition. However, this state lies at very high energies (above 18 eV), demonstrating, again, that a low-lying D_CS_ state cannot be achieved. Finally, we applied our scheme to an extended set of molecules to highlight that the three archetypes D_CS_, D_OS_, and D_mix_ are transferable models. Thus, we demonstrate the generality of our scheme for understanding doubly excited states in various systems.

In summary, we present a physically motivated definition of doubly excited character and a classification scheme able to distinguish between its limiting cases, providing a new approach to a long-standing problem. More specifically, we have highlighted challenges in the computational description of doubly excited states of various kinds, outlining the requirements for computational methods to describe them accurately. We emphasize that reproducing excitation energies is not enough to ensure the quality of a computational method to a determined system. A computation should only be deemed accurate if its wave functions and operator expectation values also comply with the reference. Nevertheless, the availability of well-defined and transferable descriptors can provide a solid basis for further discussions of the computational description of the doubly excited character. Furthermore, we hope the presented work can provide new ideas in the science surrounding doubly excited states and that the underlying physics discussed here can provide an improved language to discuss experimental results.

## Data availability

The underlying computational research data (molecular coordinates and input/output files for Q-Chem, Columbus, DFT/MRCI and TheoDORE) are available at Loughborough University's data repository at https://doi.org/10.17028/rd.lboro.22303765.

## Author contributions

Conceptualization, methodology, software: F. P.; data curation, visualization: M. T. do C.; funding acquisition and resources: M. T. do C. and M. B.; investigation, writing (original draft preparation): M. T. do C., F. P.; supervision: F. P., M. B., and J. M. T.; writing (review & editing): M. T. do C., F. P., J. M. T., M. B.

## Conflicts of interest

The authors declare no competing financial interest.

## Supplementary Material

SC-014-D2SC06990C-s001
