# Supplementary material for: Classification of doubly excited molecular electronic states
Source: Chem Sci. 2023 Mar 15;14(15):4012–26. doi: 10.1039/d2sc06990c (PMC10094316; doi:10.1039/d2sc06990c)
Supplement: SC-014-D2SC06990C-s001 [file SC-014-D2SC06990C-s001.pdf]

Electronic Supplementary Material (ESI) for Chemical Science.
This journal is © The Royal Society of Chemistry 2023

Classification of Doubly Excited Electronic States

Mariana T. do Casal[1], Josene M. Toldo[1], Mario Barbatti[1,2] and Felix Plasser[3*]

[1]*Aix-Marseille University, CNRS, Marseille, France*

[2]*Institut Universitaire de France, 75231, Paris, France*

[3]*Department of Chemistry, Loughborough University, Loughborough, LE11 3TU, U.K.*

Supporting Information

Table of Contents

S1. An alternative discussion of Ω 2

S2. 1TDM norm Ω larger than 1 3

S3. Generalization of the excitation number 4

S4. DFT/MRCI: original vs. R2018 parametrization 7

S5. DFT/MRCI vs ADC(3) 9

S6. Formaldehyde dimer 9

S6.1. ADC(3) Size Consistency 9

S7. Polyene monomers 11

S8. Cycloaddition of ethylene 15

S9. Other examples 19

S9.1. Tetracene dimer 19

S9.2. Diketopyrrolopyrrole derivatives 19

S9.3. s-Tetrazine 20

S10. References 21

S1. An alternative discussion of Ω

To provide an alternative viewpoint on the 1TDM norm Ω, we can write $|\Psi_{\mathrm{f}}\rangle$ as a combination of singly $\left|\Psi_{\mathrm{f}}^{S}\right\rangle$ and higher excited $\left|\Psi_{\mathrm{f}}^{DT\cdots}\right\rangle$ contributions with respect to $|\Psi_{\mathrm{i}}\rangle$, i.e.

$$|\Psi_{\mathrm{f}}\rangle = \left|\Psi_{\mathrm{f}}^{S}\right\rangle + \left|\Psi_{\mathrm{f}}^{DT\cdots}\right\rangle \tag{1}$$

where the singly excited contribution is defined as

$$\left|\Psi_{\mathrm{f}}^{S}\right\rangle = \sum_{pq} C_{pq}^{\mathrm{if}} q^{\dagger} p |\Psi_{\mathrm{i}}\rangle \tag{2}$$

and the higher contributions are defined analogously

$$\left|\Psi_{\mathrm{f}}^{DT\cdots}\right\rangle = \sum_{pq} C_{pqrs}^{\mathrm{if}} s^{\dagger} q^{\dagger} p r |\Psi_{\mathrm{i}}\rangle + \cdots \tag{3}$$

The coefficients in this expansion are not unique, as it is generally possible to reproduce the effect of lower excitations with higher excitation operators. However, following Ref. [1], we can choose the coefficients to maximize the overlap of $\left|\Psi_{\mathrm{f}}^{S}\right\rangle$ with $|\Psi_{\mathrm{f}}\rangle$ for any normalized set of expansion coefficients C_{pq}^{if}. For this purpose, we write the overlap between $|\Psi_{\mathrm{f}}\rangle$ and $\left|\Psi_{\mathrm{f}}^{S}\right\rangle$ as

$$\left\langle \Psi_{\mathrm{f}} \middle| \Psi_{\mathrm{f}}^{S} \right\rangle = \left\langle \Psi_{\mathrm{f}} \right| \textstyle\sum_{pq} C_{pq}^{\mathrm{if}} q^{\dagger} p \left| \Psi_{\mathrm{i}} \right\rangle = \displaystyle\sum_{pq} C_{pq}^{\mathrm{if}} D_{qp}^{\mathrm{fi}} \leq \sqrt{\sum_{pq} \left| C_{pq}^{\mathrm{if}} \right|^{2}} \sqrt{\sum_{pq} \left| D_{pq}^{\mathrm{if}} \right|^{2}} \tag{4}$$

where we have used the Cauchy-Schwarz inequality on the right-hand side. The maximum value of the overlap is obtained if C_{pq}^{if} and D_{qp}^{if} are linearly dependent. Specifically, we set $C_{pq}^{\mathrm{if}} = D_{pq}^{\mathrm{if}}/\sqrt{\Omega}$ to obtain normalized expansion coefficients. Thus, Eq. (1) reduces to

$$\left\langle \Psi_{\mathrm{f}} \middle| \Psi_{\mathrm{f}}^{S} \right\rangle = \sqrt{\Omega} \tag{5}$$

In other words, $\sqrt{\Omega}$ can be interpreted as the maximum possible overlap of Ψ_{f} with any possible function that can be constructed via single excitations from Ψ_{i}, supporting the interpretation of Ω as a measure of single-excitation character. In practical terms, an Ω value of zero means that Ψ_{f} is orthogonal to any function constructed via single excitations from Ψ_{i}, whereas higher Ω values point to enhanced contributions of single excitations.

Finally, we point out that the above derivation requires that the expansion coefficients C_{pq}^{if} are normalized but Ψ_{f}^{S} is not generally normalized. Indeed, the norm of Ψ_{f}^{S} could, in principle, be larger than one, resulting in an Ω value also larger than one. In practice, values larger than one are not usually observed, but we discuss how to construct such cases in Section S2 of the Supporting Information. In addition, Ω values slightly larger than one are encountered per construction in TDDFT computations not employing the Tamm-Dancoff approximation.[2]

S2. 1TDM norm Ω larger than 1

Within this section, we exemplify the possibility that the 1TDM norm Ω is larger than 1 in wavefunction based calculations. The case is usually not encountered in practice but, in principle, such cases can be constructed. Here we consider four different spatial orbitals, ϕ_1 to ϕ_4. Using these, one can construct two different Slater determinants $|1\bar{1}2\bar{2}\rangle$ and $|3\bar{3}4\bar{4}\rangle$ where either the first two or last two of these are doubly occupied. The wavefunctions of the initial and final states are now constructed as

$$\Psi_{\mathrm{i}} = 2^{-\frac{1}{2}}(|1\bar{1}2\bar{2}\rangle + |3\bar{3}4\bar{4}\rangle) \tag{6}$$

$$\Psi_{\mathrm{f}} = 2^{-\frac{1}{2}}(|1\bar{1}2\bar{2}\rangle - |3\bar{3}4\bar{4}\rangle) \tag{7}$$

These are normalized and orthogonal. On finds that all off-diagonal 1TDM elements vanish whereas the diagonal 1TDM elements are evaluated as

$$D_{11}^{\mathrm{if}} = D_{\bar{1}\bar{1}}^{\mathrm{if}} = D_{22}^{\mathrm{if}} = D_{\bar{2}\bar{2}}^{\mathrm{if}} = \frac{1}{2} \tag{8}$$

$$D_{33}^{\mathrm{if}} = D_{\bar{3}\bar{3}}^{\mathrm{if}} = D_{44}^{\mathrm{if}} = D_{\bar{4}\bar{4}}^{\mathrm{if}} = -\frac{1}{2} \tag{9}$$

The 1TDM norm Ω is the sum over the squares of all these elements

$$\Omega = 8 \times \left(\frac{1}{2}\right)^2 = 2 \tag{10}$$

Thus, indeed, Ω is larger than 1. Similar constructions with more partially occupied orbitals can be employed to obtain even higher values.

Analyzing this case in more detail, we find that the initial and final states both possess the same densities and, thus, we obtain for the promotion and excitation numbers

$$p = \eta = 0 \tag{11}$$

Alternatively, it is possible to compute the fraction of de-excitations according to Ref. [2].

$$P_{he} = \Omega^{-1} tr\left(\mathbf{D}^{\mathrm{if}}\mathbf{D}^{\mathrm{if}}\right) = 1 \tag{12}$$

The excited state is thus classified as being composed of 100% de-excitations highlighting, again, that this is an exotic case.

An Ω value much larger than one is not usually encountered in practical calculations but appropriate cases can be constructed. In the following we present data on the CO_2^{4+} cation with one CO distance at 1.16 Å and the other at 2.32 Å. This system possesses four approximately degenerate π-orbitals filled with four electrons, which is intended to reflect the situation sketched above.

Computations were performed in OpenMolcas[3] and wavefunctions were analyzed using the WFA[4] module. An active space containing 4 electrons in the six π/π^* orbitals was used. The results are presented in Table S1. Here, the first excited state (S_1) is used as a reference for transition and difference density matrix based properties. Crucially, we find that the transition from the S_1 state to the degenerate S_2 state is indeed characterized by Ω = 1.988. However, the presented case is clearly far from typical calculations. First, one has to realize that the large Ω value is only observed for the $S_1 \rightarrow S_2$ transition and not for any transition involving the ground state. Second, we find that all states possess the same 1DMs, as indicated by vanishing p and η values. As such, we highlight that the meaning of all three descriptors – Ω, p and η – has to be rethought in the cases of *initial* states with many open shells.

Table S1: Analysis of excited states of the CO_2^{4+} cation with one broken bond illustrating the possibility of obtaining Ω values above 1. The S_1 state is used as *initial* state for the transition and difference density matrix based properties.

State	ΔE	f	Ω	PR_{NTO}	$n_{u,nl}$	y_0	y_1	p	η
S_0	-4.474	0.000	0.000	-	3.999	0.985	0.985	0.087	0.005
S_1	0.000	0.000	-	-	3.999	0.983	0.983	-	-
S_2	0.000	0.000	1.988	4.000	3.999	0.983	0.983	0.000	0.000
S_3	0.002	0.000	0.000	-	4.000	0.994	0.994	0.027	0.000
S_4	0.002	0.000	0.000	-	4.000	0.994	0.994	0.027	0.000

S3. Generalization of the excitation number

The excitation number between two states described via individual Slater determinants was defined as

$$\eta = n - \sum_{jk}^{\text{occ}} \left| S_{jk}^{\text{if}} \right|^2 \qquad (13)$$

as described in the main text.[5] Eq. (13) can directly be applied if the initial and final state are both described by single Slater determinant. In the following, we want to discuss how to generalize this to arbitrary wavefunctions. There is no unique way to generalize Eq. (13) and we will evaluate several options. We first realize that the summation in Eq. (13) corresponds to the squared Frobenius norm of the occupied-occupied block of the mixed overlap matrix $\mathbf{S}_{\text{if}}$

$$\eta = n - \|\mathbf{S}_{\text{if}}\|_F^2 = n - \text{tr}(\mathbf{S}_{\text{if}}\mathbf{S}_{\text{if}}^T) \tag{14}$$

One furthermore realizes that the mixed overlap matrix is obtained from the MO coefficients and the AO overlap matrix as $\mathbf{S}_{\text{if}} = \mathbf{C}_{\text{i}}^T \mathbf{S}_{\text{AO}} \mathbf{C}_{\text{f}}$ yielding

$$\eta = n - \text{tr}\left(\mathbf{C}_{\text{i}}^T \mathbf{S}_{\text{AO}} \mathbf{C}_{\text{f}} \mathbf{C}_{\text{f}}^T \mathbf{S}_{\text{AO}} \mathbf{C}_{\text{i}}\right) = n - 0.5\ \text{tr}\left(\mathbf{D}_{\text{ii,AO}} \mathbf{S}_{\text{AO}} \mathbf{D}_{\text{ff,AO}} \mathbf{S}_{\text{AO}}\right) \tag{15}$$

where we introduced the spin-traced density matrices $\mathbf{D}_{\text{ii,AO}} = 2\mathbf{C}_{\text{i}}\mathbf{C}_{\text{i}}^T$ and $\mathbf{D}_{\text{ff,AO}} = 2\mathbf{C}_{\text{f}}\mathbf{C}_{\mathbf{f}}^T$. Introduction of the density matrices as shown in Eq. (15) only holds for single-determinantal wavefunctions. However, we can use the r.h.s. of Eq. (15) as a definition of a generalized excitation number where we can now also include arbitrary density matrices. To obtain a simpler form, we transform the r.h.s. of Eq. (15) into an orthogonal basis (meaning that the overlap matrix vanishes). This yields the form

$$\eta = n - 0.5\ \text{tr}(\mathbf{D}_{\text{ii}}\mathbf{D}_{\text{ff}}) \tag{16}$$

Excitation numbers for hexatriene computed with this formula are presented in the column "η Eq. (16)" in Table S2. The obtained values are close to three for the singly excited B_u states and close to four for the doubly excited 2^1A_g state. One realizes that these values are too high by about two compared to the idealized values of singly and doubly excited character. This value is reflected by the number of unpaired electrons (n_u) of the ground state. To understanding this problem, one first realizes that the expression in Eq. (16) does not vanish if one sets i=f. Indeed, this produces

$$\eta = n - 0.5\ \text{tr}(\mathbf{D}_{\text{ii}}\mathbf{D}_{\text{ii}}) = \text{tr}(\mathbf{D}_{\text{ii}} - 0.5\ \mathbf{D}_{\text{ii}}\mathbf{D}_{\text{ii}}) \tag{17}$$

which is not equal to zero unless all orbitals are doubly occupied. Indeed, Eq. (6) is related to Takatsuka et al.'s definition of the distribution of odd electrons.[6] An additional change has to be made in order to assure that the expression vanishes in the case i=f. For this purpose, we replace the number *n* in Eq. (17) with a number n_{eff} to assure that the excitation number for the i=f case vanishes. We write

$$\eta = n_{\text{eff}} - 0.5\ \text{tr}(\mathbf{D}_{\text{ii}}\mathbf{D}_{\text{ff}}) \tag{18}$$

Two definitions of n_{eff} were investigated

$$n_{\text{eff}} = \left(\|\mathbf{D}^{\text{ii}}\|^2 + \|\mathbf{D}^{\text{ff}}\|^2\right)/4 \tag{19}$$

$$n_{\text{eff}} = \max\left(\|\mathbf{D}^{\text{ii}}\|^2, \|\mathbf{D}^{\text{ff}}\|^2\right)/2 \tag{20}$$

Both definitions vanish for the i=f case, are symmetric with respect to an interchange of i and f and reduce to Eq. (3) if the density matrices derive from a single Slater determinant with doubly occupied orbitals. Results using these definitions are shown in the last two columns of Table S2. Only the combination of Eqs (18) and (20) leads the expected results: singly excited character of the B_u states and partially doubly excited character for 2^1A_g. We, therefore, use this definition of η in the main text. Note that this is equivalent to the formula

$$\eta = 0.5\left(\max\left[\sum_p \left(n_p^{\mathrm{i}}\right)^2, \sum_q \left(n_q^{\mathrm{f}}\right)^2\right] - \sum_{pq} n_p^{\mathrm{i}} n_q^{\mathrm{f}} \left|S_{pq}^{\mathrm{if}}\right|^2\right) \tag{21}$$

Table S2: Analysis of the excited states of hexatriene computed at the ADC(3) level. A comparison of different definitions of the excitation number as defined in the text above is presented.

State	ΔE	*f*	Ω	n_u	η Eq. (16)	η Eqs (18,19)	η Eqs (18,20)
S_0	0.000	-	-	1.97	-	-	-
1^3B_u	1.807	0.000	0.836	3.89	2.97	0.34	1.04
2^1A_g	3.323	0.000	0.175	4.23	3.62	0.81	1.64
1^1B_u	4.206	2.651	0.846	3.86	2.87	0.36	0.94

S4. DFT/MRCI: original vs. R2018 parametrization

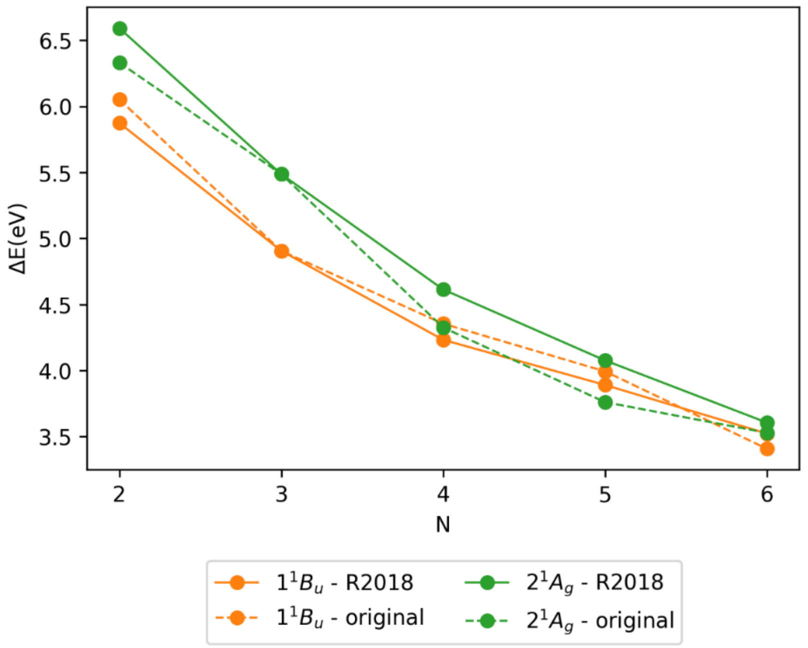

Figure S1. Vertical excitation energies (eV) of polyenes with N double bonds of states 1^1B_u (yellow) and 2^1A_g (green) at DFT/MRCI def2-TZVP level with R2018 (solid line) and original parametrization (dashed line).

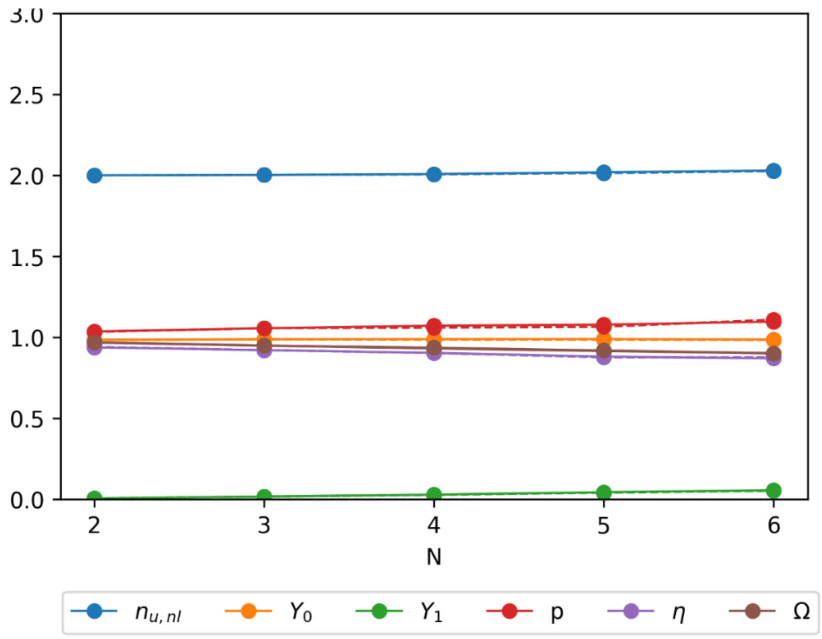

Figure S2. Wavefunction descriptors of polyenes with N double bonds in 1^1B_u state at DFT/MRCI def2-TZVP with R2018 (solid line) and original (dashed line) parametrization.

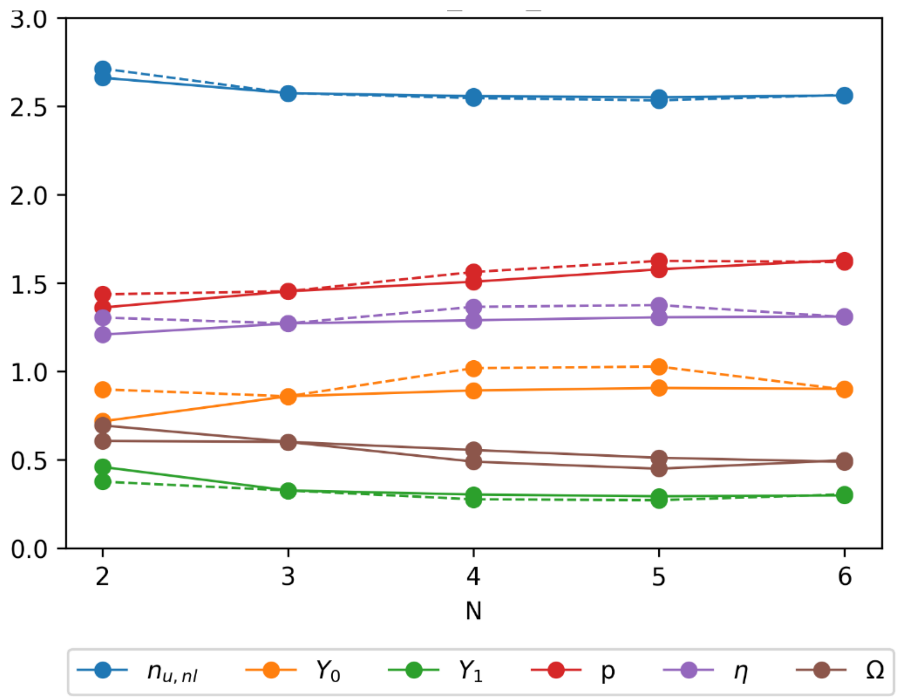

Figure S3. Wavefunction descriptors of of polyenes with N double bonds in 2^1A_g state at DFT/MRCI def2-TZVP with R2018 (solid line) and original (dashed line) parametrization.

S5. DFT/MRCI vs ADC(3)

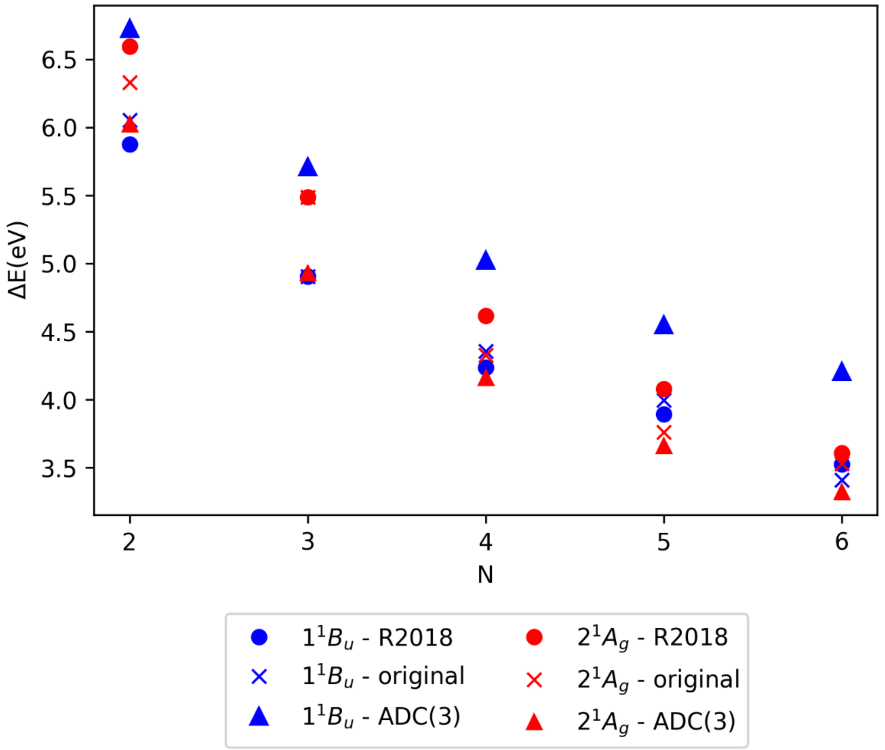

Figure S4. Excitation energies of polyenes with N double bonds in eV of the 1^1B_u and 2^1A_g states at DFT/MRCI with the original and R2018 parametrizations and at ADC(3) level.

S6. Formaldehyde dimer

S6.1. ADC(3) Size Consistency

ADC(3) is size-consistent with respect to the excitation energies, transition moments, and properties of individual states.[7] However, one could extend the definition of size-consistency to multiply excited states and ask if the excitation energy of the doubly excited state of a non-interacting dimer is twice the excitation energy of the singly excited state of the monomer. This cannot be achieved by a method with a fixed number of excitations, such as ADC(3). Indeed, within ADC(3), singly excited states are described in third order but doubly excited states are only in the first order.[7] The lack of this extended type of size consistency explains the difference in excited-state energies and is also seen in the $n_{u,nl}$ (2.46 vs 4.05) and p (1.15 vs 2.00) descriptors, shown in Table 1 of the main text, highlighting that neither doubles appropriately. Indeed, the doubly excited state show values smaller than expected. Since $n_{u,nl}$ and p reflect electron correlation and orbital relaxation effects, lower values than expected mean that the doubly excited state can take advantage of less electron correlation and orbital relaxation compared to the singly excited state.

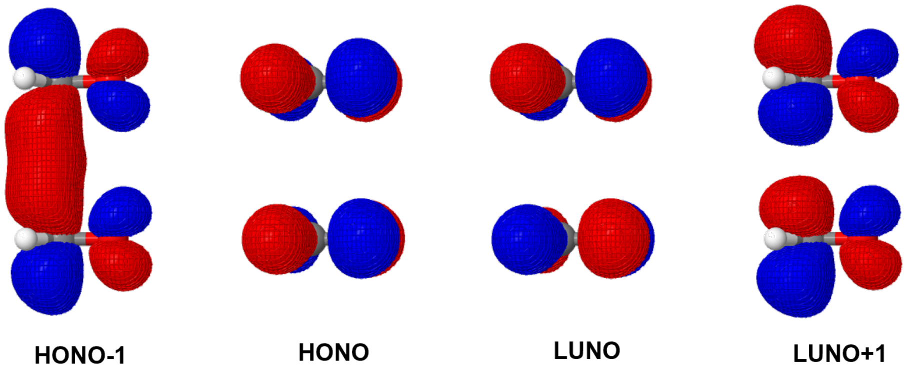

Figure S5. Natural orbitals of formaldehyde dimer of 2^1A_1. HONO and LUNO stands for the highest and lowest occupied natural orbitals.

S7. Polyene monomers

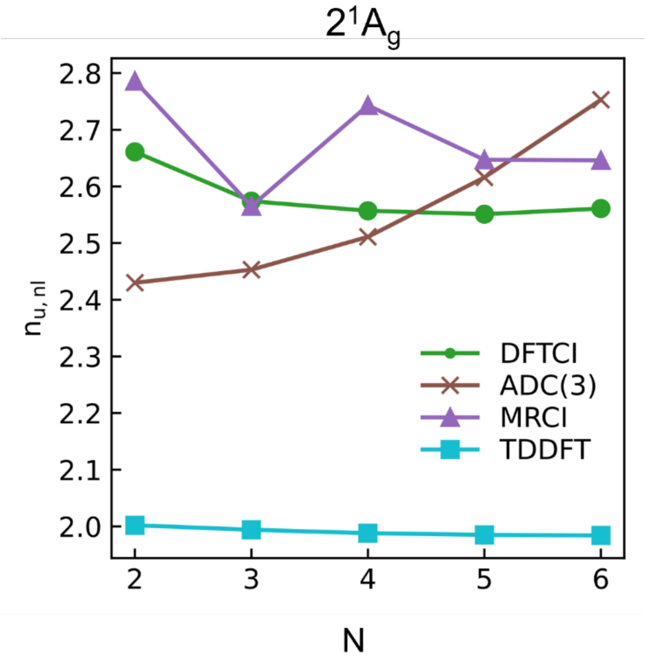

Figure S6. Number of unpaired electrons ($n_{u,nl}$) along the systematic increase of the number of double bonds (N) of polyenes in states 2^1Ag at TDDFT/BHLYP, ADC(3), DFT/MRCI and MRCI levels

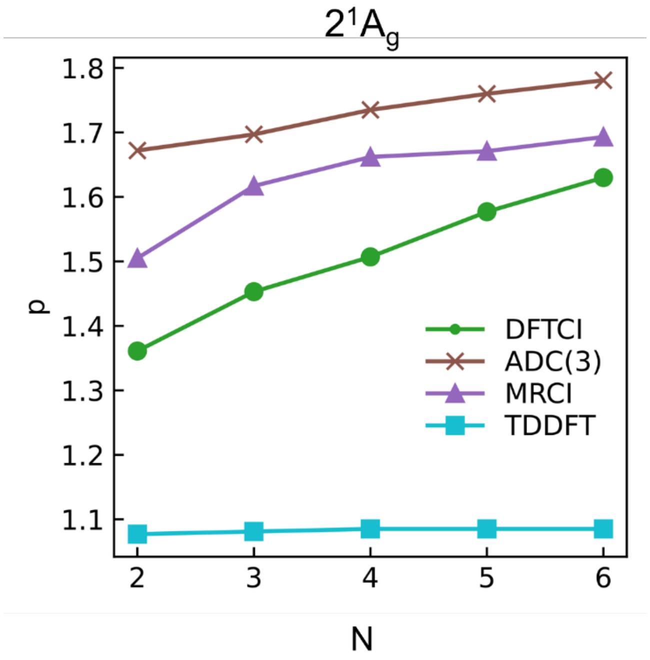

Figure S7. Promotion number (p) along the systematic increase of the number of double bonds (N) of polyenes in states 2^1Ag at TDDFT/BHLYP, ADC(3), DFT/MRCI and MRCI levels.

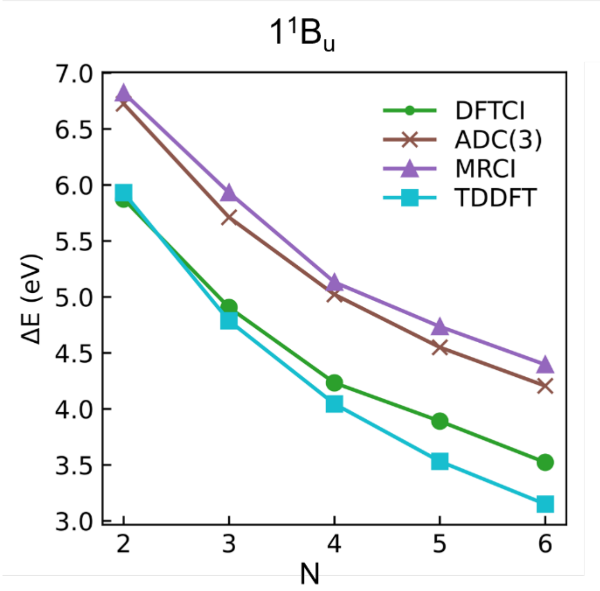

Figure S8. Excitation energies (in eV) along the systematic increase of the number of double bonds (N) of polyenes in states 1^1B_u at TDDFT/BHLYP, ADC(3), DFT/MRCI and MRCI levels.

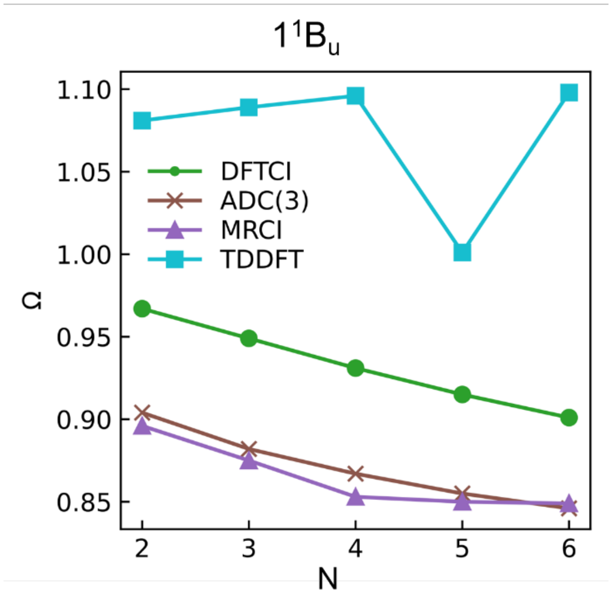

Figure S9. Ω values along the systematic increase of the number of double bonds (N) of polyenes in states 1^1B_u at TDDFT/BHLYP, ADC(3), DFT/MRCI and MRCI levels.

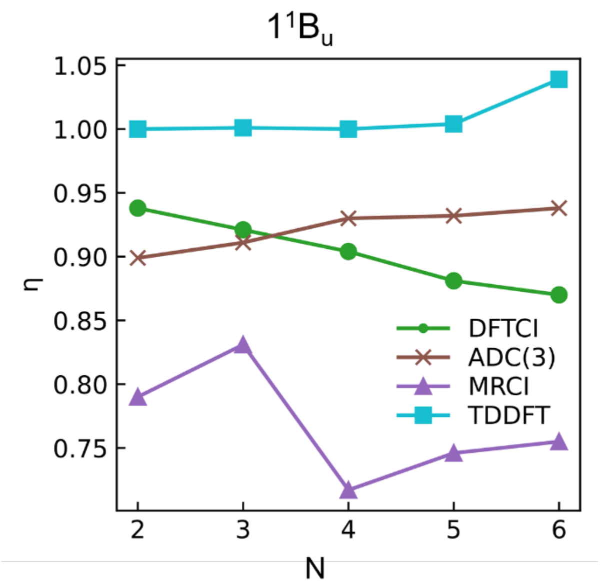

Figure S10. Excitation number (η) along the systematic increase of the number of double bonds (N) of polyenes in states 1^1B_u at TDDFT/BHLYP, ADC(3), DFT/MRCI and MRCI levels.

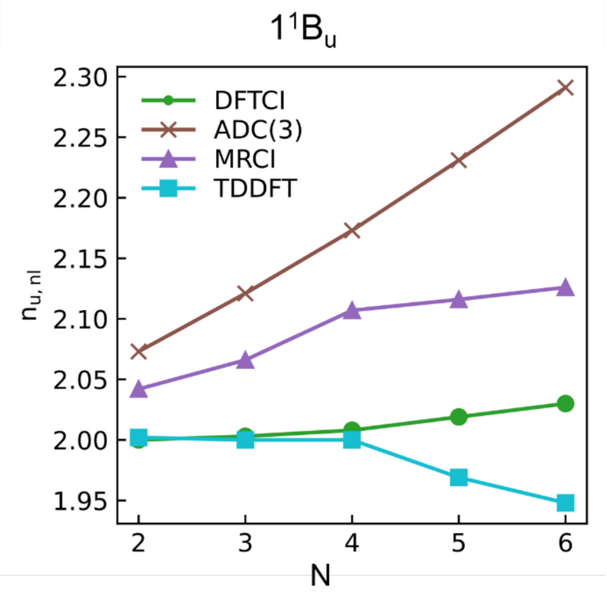

Figure S11. Number of unpaired electrons ($n_{u,nl}$) along the systematic increase of the number of double bonds (N) of polyenes in states 1^1B_u at TDDFT/BHLYP, ADC(3), DFT/MRCI and MRCI levels.

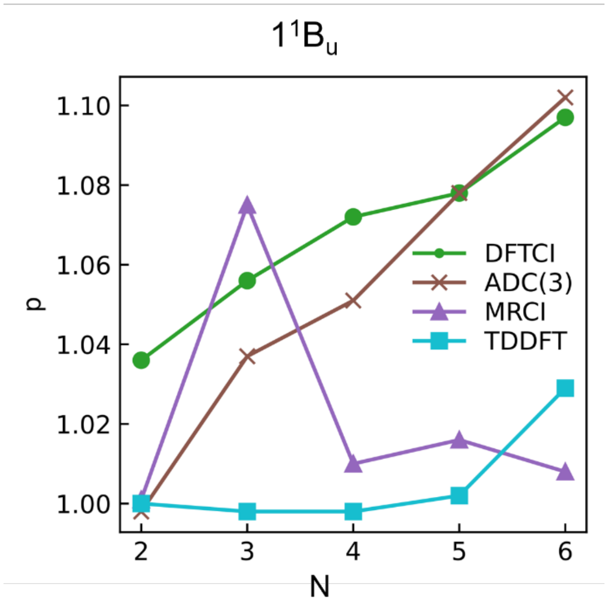

Figure S12. Promotion number (p) along the systematic increase of the number of double bonds (N) of polyenes in states 1^1B_u at TDDFT/BHLYP, ADC(3), DFT/MRCI and MRCI levels.

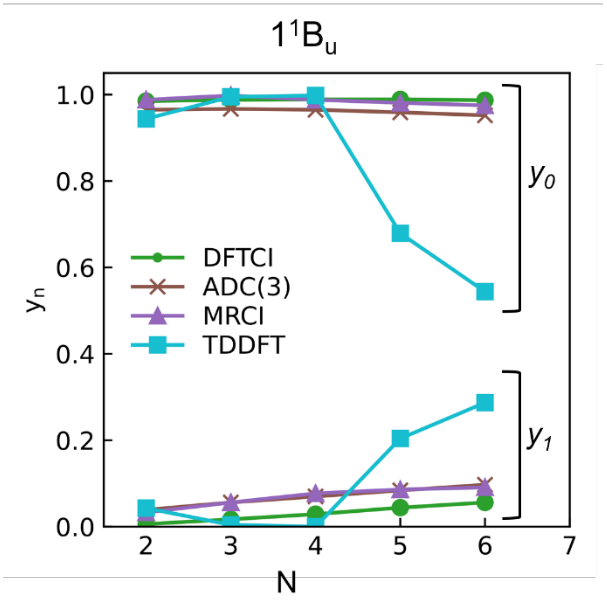

Figure S13. Occupation numbers of the lowest unoccupied natural orbital (LUNO, y_0) and LUNO+1 (y_1) along the systematic increase of the number of double bonds (N) of polyenes in states 1^1B_u at TDDFT/BHLYP, ADC(3), DFT/MRCI and MRCI levels.

S8. Cycloaddition of ethylene

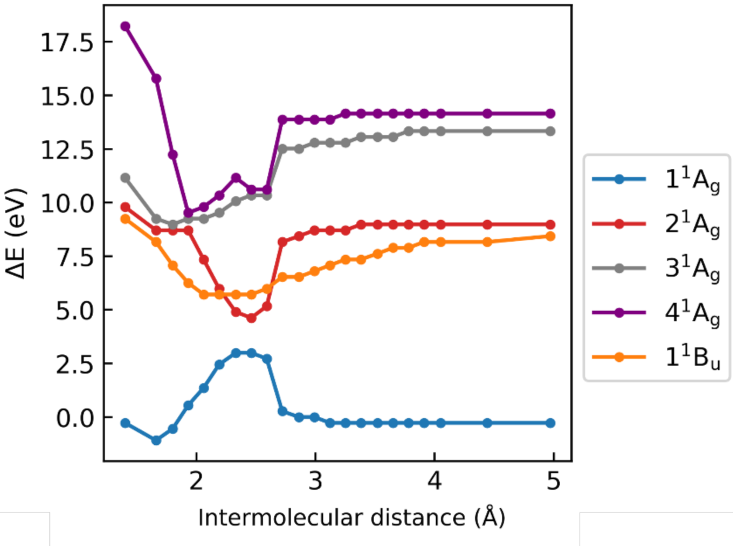

Figure S14. Excitation energies (in eV) of the first four singlet A_g states and the $1B_u$ states as the intermolecular distance between the ethylenes increases.

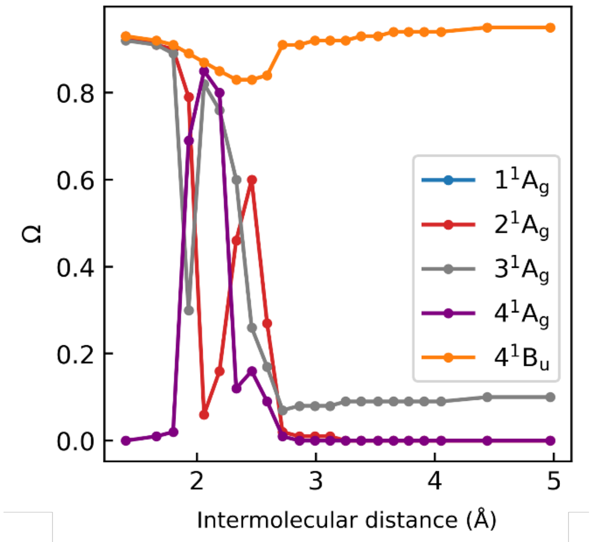

Figure S15. Ω of the first four singlet A_g states and the $1B_u$ state as the intermolecular distance between the ethylenes increases.

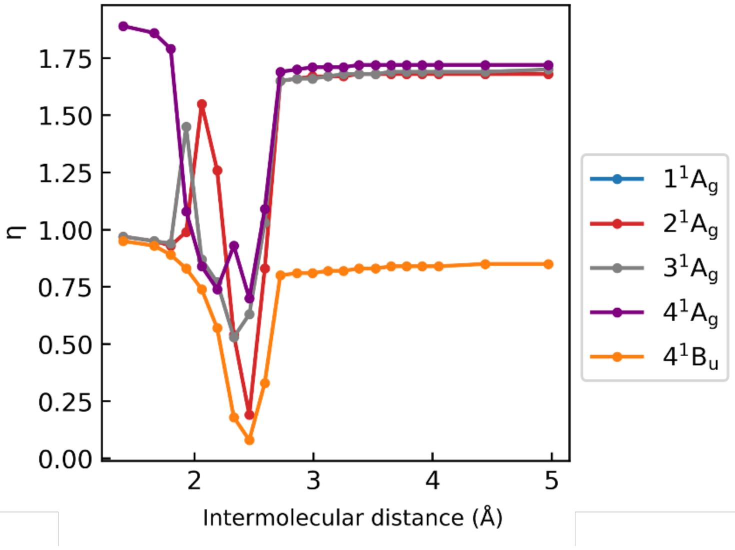

Figure S16. Excitation number (η) of the first four singlet A_g states and the $1B_u$ state as the intermolecular distance between the ethylenes increases.

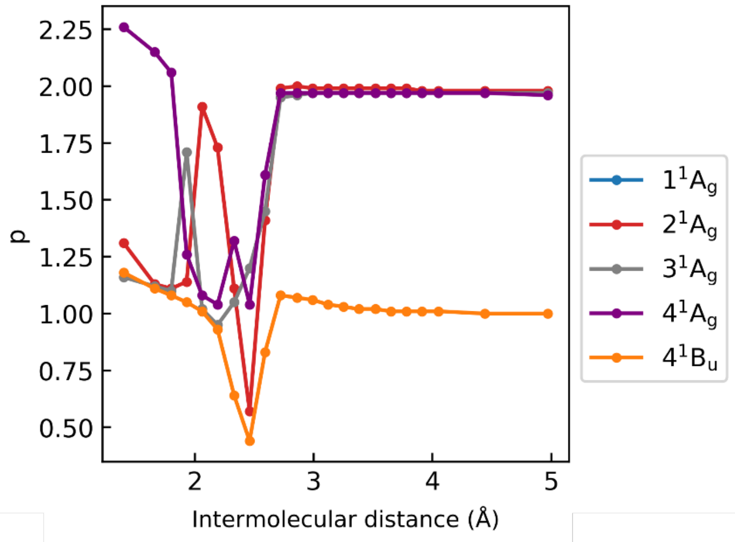

Figure S17. Promotion number (*p*) of the first four singlet A_g states and the $1B_u$ state as the intermolecular distance between the ethylenes increases.

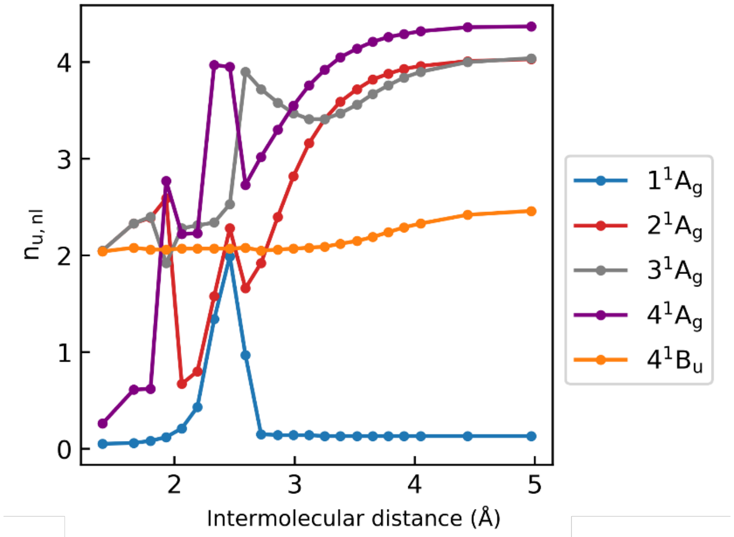

Figure S18. Number of unpaired electrons ($n_{u,nl}$) of the first four singlet A_g states and the $1B_u$ state as the intermolecular distance between the ethylenes increases.

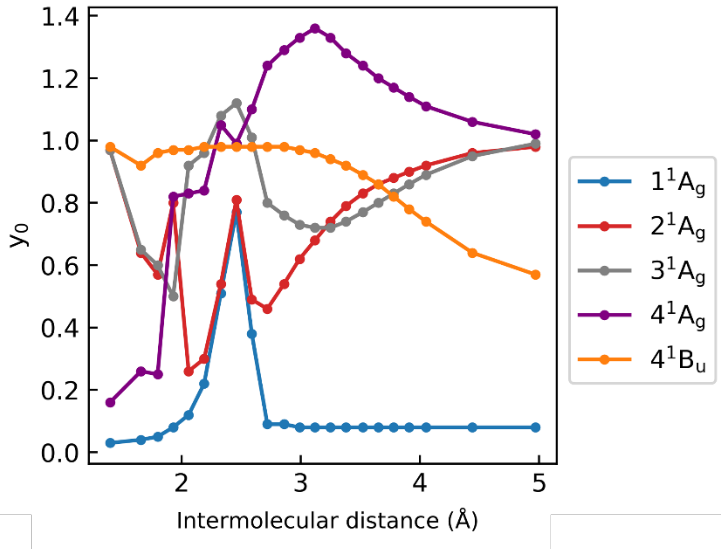

Figure S19. Occupation number of the lowest unoccupied natural orbital (*y_0*) of the first four singlet A_g states and the $1B_u$ state as the intermolecular distance between the ethylenes increases.

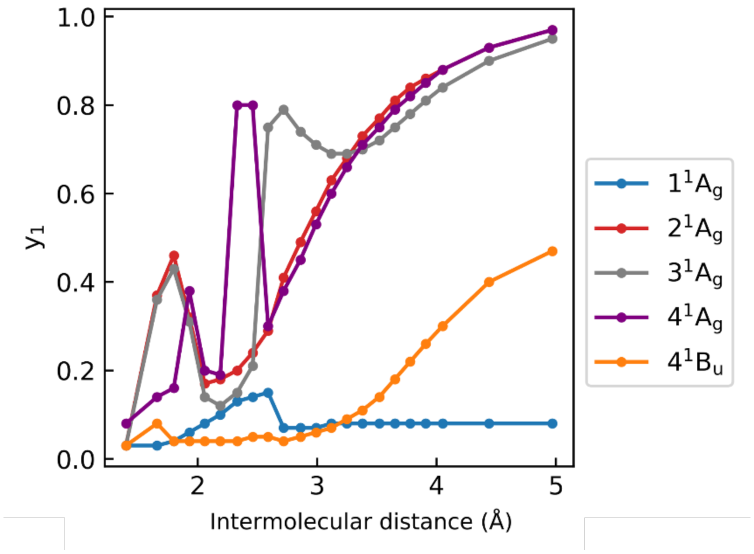

Figure S20. Occupation number of the lowest unoccupied natural orbital +1 (*y_1*) of the first four singlet A_g states and the $1B_u$ state as the intermolecular distance between the ethylenes increases.

S9. Other examples

S9.1. Tetracene dimer

Table S3 Excitation energy (ΔE in eV), oscillator strength (*f*), and wave function descriptors of the lowest excited singlet states of the stacked tetrazine dimer at 5 Å apart computed at the DFT/MRCI/def2-SV(P) level.

State	ΔE (eV)	*f*	Ω	PR_{NTO}	$n_{u,nl}$	y_0	y_1	*p*	*η*
1^1A_g	0.00	0.000			0.230	0.119	0.117	-	-
1^3A_g	1.11				1.955	0.344	0.342	0.817	0.979
1^3A_u	1.11				4.038	0.997	0.937	0.817	0.979
2^1A_g	2.16	0.000	0.000	2.435	4.046	1.003	0.949	1.567	2.095
1^3B_g	2.25				2.515	0.576	0.500	1.564	2.165
1^1B_g	2.30	0.000	0.894	2.028	2.520	0.681	0.419	0.791	1.057
1^1B_u	2.35	0.192	0.905	2.221	2.609	0.576	0.537	0.809	1.070
1^3B_u	2.56				2.519	0.540	0.536	0.782	1.102
2^3A_g	2.56				4.041	0.991	0.990	0.782	1.102
2^3A_u	2.61				1.958	0.363	0.324	1.565	2.176
2^3B_g	2.78				2.423	0.561	0.491	0.817	0.980
2^1B_u	2.78	0.000	0.820	1.999	2.429	0.545	0.507	0.743	1.044
2^3B_u	2.79				2.427	0.528	0.525	0.736	1.081
2^1B_g	2.80	0.000	0.820	1.851	2.394	0.656	0.398	0.746	1.045
1^1A_u	2.94	0.000	0.174	4.461	4.009	0.969	0.855	1.524	1.878

S9.2. Diketopyrrolopyrrole derivatives

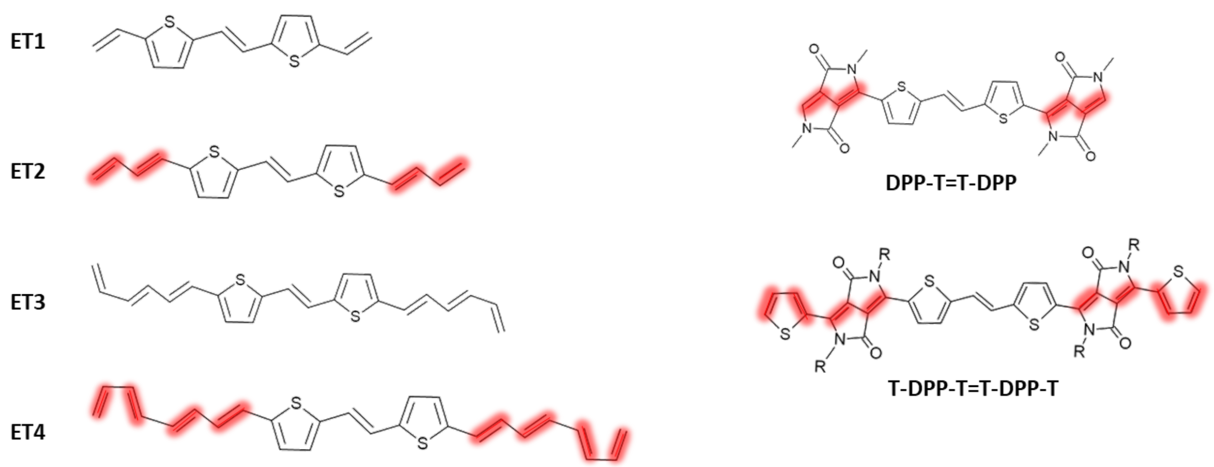

Figure 21 Molecular structure of the diketopyrrolopyrroles derivatives.

Table S4 Excitation energy (ΔE in eV), oscillator strength (*f*), and wave function descriptors of the lowest excited states of diketopyrrolpyrrole derivatives (see Figure 21) computed at the DFT/MRCI def2-SV(P) level. From Ref. 8.

	Sym	EE	Ω	*p*	η	$n_{u,nl}$	y_0	y_1
**T=T**	$2A_g$	4.428	0.611	1.505	1.235	2.559	0.839	0.321
	$1B_u$	3.600	0.927	1.095	0.917	2.017	0.984	0.040
**ET1-T=T-ET1**	$2A_g$	3.425	0.56	1.608	1.257	2.558	0.758	0.391
	$1B_u$	2.971	0.904	1.127	0.907	2.037	0.979	0.060
**ET1-T=T-ET2**	$2A_g$	2.837	0.501	1.728	1.289	2.585	0.786	0.371
	$1B_u$	2.595	0.883	1.160	0.890	2.067	0.973	0.081
**ET1-T=T-ET3**	$2A_g$	2.371	0.44	1.857	1.320	2.627	0.783	0.378
	$1B_u$	2.297	0.861	1.200	0.871	2.110	0.964	0.104
**ET1-T=T-ET4**	$2A_g$	2.005	0.376	1.978	1.351	2.716	0.791	0.391
	$1B_u$	2.070	0.835	1.243	0.848	2.166	0.952	0.129
**DPP-T=T-DPP**	$2A_g$	1.846	0.372	1.930	1.329	2.617	0.956	0.236
	$1B_u$	1.923	0.758	1.410	0.941	2.135	0.946	0.129
**T-DPP-T=T-DPP-T**	$2A_g$	1.566	0.331	1.966	1.340	2.690	0.864	0.320
	$1B_u$	1.658	0.814	1.251	0.854	2.157	0.938	0.134
**T-DPP-T**	2A	2.322	0.884	1.128	0.916	2.025	0.991	0.047
	3A	3.248	0.57	1.500	1.227	2.636	0.725	0.574

S9.3. s-Tetrazine

Table S5 Excitation energy (ΔE in eV), oscillator strength (*f*), and wave function descriptors of the lowest excited states of s-tetrazine computed at the DFT/MRCI/def2-TZVP level.

State	ΔE (eV)		*f*	Ω	PR_{NTO}	$n_{u,nl}$	y_0	y_1	*p*	*η*
$1\,^1A_g$	0.00		-	-	-	0.02	0.05	0.03	-	-
$1\,^3B_{1u}$	2.09	H → L	-	-	1.01	2.01	1.00	0.03	2.00	1.06
$1\,^1B_{1u}$	2.59	H→ L	0.011	0.93	2.78	2.01	1.02	0.03	1.05	0.96
$1\,^3B_{2u}$	3.48	H-2 H → L L	-	-	-	2.04	0.93	0.09	1.29	0.68
$1\,^3B_{3u}$	3.53	H-5 H → L L	-	-	-	2.20	0.72	0.29	1.18	0.47
$1\,^3Au$	3.78	H H → L L+1	-	-	-	2.05	0.96	0.10	1.66	0.82
$1\,^1Au$	4.03	H H → L L+1	0.000	0.90	1.06	2.04	0.99	0.08	1.13	1.00
$2\,^3B_{3u}$	4.22	H-2 H H → L L L+1	-	-	-	2.28	0.72	0.32	0.53	0.33
$1\,^1B_{2u}$	4.31	H-2 H → L L	0.060	0.87	2.19	2.23	0.82	0.24	1.12	0.98
$1\,^3B_{3g}$	4.72	H-1 H → L L	-		-	2.07	0.94	0.08	1.89	0.84
$2\,^1A_g$	5.11	H H → L L	0.000	0.00	-	0.14	0.15	0.04	2.06	1.89
$1\,^1B_{3g}$	5.25	H-1 H → L L	0.000	0.80	2.61	2.10	1.07	0.09	1.16	1.08
$2\,^3A_u$	5.29	H-3 H → L L	-	0.01	-	2.02	0.99	0.06	2.01	0.98
$1\,^3B_{2g}$	5.42	H-4 H → L L	-	0.01	-	2.15	0.92	0.22	1.79	0.61
$2\,^1A_u$	5.60	H-3 H → L L	0.000	0.90	1.01	2.02	1.02	0.04	1.08	0.99

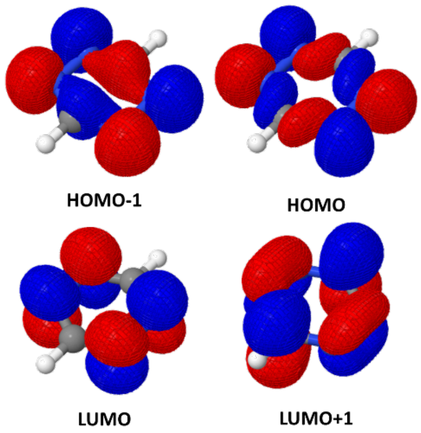

Figure 22 Molecular orbitals of s-tetrazine.

S10. References

1 S. Klaiman and L. S. Cederbaum, *J. Chem. Phys.*, 2014, **141**, 194102.

2 P. Kimber and F. Plasser, *Phys. Chem. Chem. Phys.*, 2020, **22**, 6058–6080.

3 I. Fdez. Galván, M. Vacher, A. Alavi, C. Angeli, F. Aquilante, J. Autschbach, J. J. Bao, S. I. Bokarev, N. A. Bogdanov, R. K. Carlson, L. F. Chibotaru, J. Creutzberg, N. Dattani, M. G. Delcey, S. S. Dong, A. Dreuw, L. Freitag, L. M. Frutos, L. Gagliardi, F. Gendron, A. Giussani, L. González, G. Grell, M. Guo, C. E. Hoyer, M. Johansson, S. Keller, S. Knecht, G. Kovačević, E. Källman, G. Li Manni, M. Lundberg, Y. Ma, S. Mai, J. P. Malhado, P. Å. Malmqvist, P. Marquetand, S. A. Mewes, J. Norell, M. Olivucci, M. Oppel, Q. M. Phung, K. Pierloot, F. Plasser, M. Reiher, A. M. Sand, I. Schapiro, P. Sharma, C. J. Stein, L. K. Sørensen, D. G. Truhlar, M. Ugandi, L. Ungur, A. Valentini, S. Vancoillie, V. Veryazov, O. Weser, T. A. Wesołowski, P. O. Widmark, S. Wouters, A. Zech, J. P. Zobel and R. Lindh, *J. Chem. Theory Comput.*, 2019, **15**, 5925–5964.

4 F. Plasser, S. A. Mewes, A. Dreuw and L. González, *J. Chem. Theory Comput.*, 2017, **13**, 5343–5353.

5 G. M. J. Barca, A. T. B. Gilbert and P. M. W. Gill, *J. Chem. Theory Comput.*, 2018, **14**, 9–13.

6 K. Takatsuka, T. Fueno and K. Yamaguchi, *Theor. Chim. Acta*, 1978, **48**, 175–183.

7 A. Dreuw and M. Wormit, *WIREs Comput. Mol. Sci.*, 2015, **5**, 82–95.

8 M. T. do Casal, J. M. Toldo, F. Plasser and M. Barbatti, *Phys. Chem. Chem. Phys.*, 2022, 23279–23288.
